# Heat Stress-Induced PI3K/mTORC2-Dependent AKT Signaling Is a Central Mediator of Hepatocellular Carcinoma Survival to Thermal Ablation Induced Heat Stress

**DOI:** 10.1371/journal.pone.0162634

**Published:** 2016-09-09

**Authors:** Scott M. Thompson, Matthew R. Callstrom, Danielle E. Jondal, Kim A. Butters, Bruce E. Knudsen, Jill L. Anderson, Karen R. Lien, Shari L. Sutor, Ju-Seog Lee, Snorri S. Thorgeirsson, Joseph P. Grande, Lewis R. Roberts, David A. Woodrum

**Affiliations:** 1 Department of Radiology, Mayo Clinic College of Medicine, Rochester, MN, United States of America; 2 Department of Laboratory Medicine and Pathology, Mayo Clinic College of Medicine, Rochester, MN, United States of America; 3 Genomics Research Center, Mayo Clinic College of Medicine, Rochester, MN, United States of America; 4 Division of Cancer Medicine, University of Texas MD Anderson Cancer Center, Houston, TX, United States of America; 5 Center for Cancer Research, National Cancer Institute, Bethesda, Maryland, United States of America; 6 Division of Gastroenterology and Hepatology, Mayo Clinic College of Medicine, Rochester, MN, United States of America; Universitatsmedizin Greifswald, GERMANY

## Abstract

Thermal ablative therapies are important treatment options in the multidisciplinary care of patients with hepatocellular carcinoma (HCC), but lesions larger than 2–3 cm are plagued with high local recurrence rates and overall survival of these patients remains poor. Currently no adjuvant therapies exist to prevent local HCC recurrence in patients undergoing thermal ablation. The molecular mechanisms mediating HCC resistance to thermal ablation induced heat stress and local recurrence remain unclear. Here we demonstrate that the HCC cells with a poor prognostic hepatic stem cell subtype (Subtype HS) are more resistant to heat stress than HCC cells with a better prognostic hepatocyte subtype (Subtype HC). Moreover, sublethal heat stress rapidly induces phosphoinositide 3-kinase (PI3K)/mammalian target of rapamycin (mTOR) dependent-protein kinase B (AKT) survival signaling in HCC cells *in vitro* and at the tumor ablation margin *in vivo*. Conversely, inhibition of PI3K/mTOR complex 2 (mTORC2)-dependent AKT phosphorylation or direct inhibition of AKT function both enhance HCC cell killing and decrease HCC cell survival to sublethal heat stress in both poor and better prognostic HCC subtypes while mTOR complex 1 (mTORC1)-inhibition has no impact. Finally, we showed that AKT isoforms 1, 2 and 3 are differentially upregulated in primary human HCCs and that overexpression of AKT correlates with worse tumor biology and pathologic features (AKT3) and prognosis (AKT1). Together these findings define a novel molecular mechanism whereby heat stress induces PI3K/mTORC2-dependent AKT survival signaling in HCC cells and provide a mechanistic rationale for adjuvant AKT inhibition in combination with thermal ablation as a strategy to enhance HCC cell killing and prevent local recurrence, particularly at the ablation margin.

## Introduction

Hepatocellular carcinoma (HCC) is a deadly, increasingly common disease for which better treatments are needed [1–5]. Image-guided percutaneous thermal ablative therapies have greatly expanded treatment options for patients with early-stage HCC, achieving short-term outcomes similar to surgery with less morbidity [6–8]. However, as tumor size increases, thermal ablation of HCC is plagued by high local recurrence and intrahepatic tumor progression rates (up to 75% at 5 years), particularly for tumors beyond 2-3cm in size. Additionally, overall survival of these patients remains poor [6–14]. Of concern, there is evidence for rapid, aggressive intrasegmental HCC recurrence following thermal ablation and currently no adjuvant therapies exist to help prevent recurrence [2, 13–18]. The ablation margin is the most common site for HCC local tumor recurrence, suggesting incomplete treatment resulting from sublethal heat stress or increase tumoral resistance [19–21].

Coagulation necrosis has been considered the primary mechanism of cell death from thermal ablation. However, given the wide variation in temperatures seen at the ablation margin in experimental studies, uniform achievement of at least 50°C throughout the tumor volume is unlikely and some regions receive a lower than desired thermal dose [22, 23]. Consequently, rapid coagulation necrosis of the entire tumor volume may not be achieved and the injured HCC cells may or may not progress to irreversible cell injury depending on the balance between pro-death versus pro-survival mechanisms. More recent studies have demonstrated that both regulated and non-regulated cell death mechanisms mediate heat stress-induced HCC cell killing in a thermal-dose dependent manner and that the mechanisms vary between hepatocytes and different HCC cell lines [24]. Moreover, apoptosis is a significant mechanism of cell death at the HCC tumor ablation margin [24].

Unfortunately, the clinical use of thermal ablation for HCC has outpaced the understanding of the basic molecular mechanisms mediating HCC-specific response to heat stress, particularly at the ablation margin where tumor recurrence remains a challenging problem [25–27]. Application of modern genomics and molecular biology techniques has showed that HCC is a heterogeneous malignancy comprised of different molecular prognostic subtypes and driven by diverse oncogenic signaling pathways [28–31]. However, the impact of the biological heterogeneity of HCC on therapeutic response to heat stress remains unknown. As such, fundamental basic and translational questions remain unanswered regarding the impact of tumor specific HCC biology on mechanisms of cell survival and alterations in oncogenic signaling in response to heat stress. Consequently, there remains a critical need to delineate molecular mechanisms mediating HCC local recurrence, particularly at the ablation margin, in order to identify therapeutic targets and develop adjuvant therapeutic strategies for enhancing thermal ablation-induced HCC cell killing.

The aims of the present study were three-fold: first, to characterize the molecular prognostic subtype of two phenotypically distinct HCC cell lines used for testing thermal ablation hypotheses and determine their thermal sensitivity using clinically relevant experimental heat stress conditions; second, to identify molecular mechanisms that promote HCC cell survival to heat stress as potential candidate adjuvant therapeutic targets, with particular attention to the PI3K-AKT-mTOR pathway; and third, to investigate the biologic and prognostic significance of candidate pathways to human HCC. The experiments herein provide evidence that sublethal heat stress induced PI3K/mTORC2-dependant AKT signaling is a central mediator of HCC cell survival to thermal ablation induced heat stress in diverse molecular prognostic subtypes of HCC, thereby providing a mechanistic basis for adjuvant AKT inhibition as a therapeutic strategy to enhance thermal ablation induced HCC cell killing and prevent local recurrence, particularly at the ablation margin.

## Materials and Methods

### Ethics Statement

The microarray gene expression analysis of human HCCs reported in this study was approved by the Institutional Review Boards (IRB) of the Mayo Clinic, the Cancer Institute of the Chinese Academy of Medical Sciences, the University of Leuven and the US National Cancer Institute. Data from this patient population have been previously published [29, 32–35]. Deidentified anonymized samples were either from patients who had provided verbal or written informed consent or were surgical waste tissues from deceased patients, which were used with IRB approval. Written informed consent from a next of kin was not obtained in the case of deceased patients and the deceased patients did not constitute a vulnerable population. Verbal consent was the standard requirement of the Mayo Clinic Institutional Review Board at the time the Mayo Hepatobiliary Neoplasia Biorepository was initiated in 2001. Verbal consent was obtained from patients and documented in the patient medical record as instructed and approved by the Mayo Clinic IRB from 2001 to 2003. Beginning in 2003, the Mayo Clinic IRB required written informed consent and this was obtained and the signed consent form was scanned into each patient’s electronic medical record.

### Materials

#### Cell Lines

Clone9, N1S1, Hep3B, HepG2, PLC/PRF/5, SNU-387, SNU-423, SNU-449, SNU-475 (ATCC, Manassas, VA), AS30D (DSMZ, Braunschweig, Germany) and HuH-7 (JCRB Cell Bank, Japan) cell lines were cultured according to supplier recommendations.

#### Inhibitors

NVP-BEZ235, selumetinib (AZD6244), MK-2206 and rapamycin were purchased from Selleck Chemicals (Houston, TX); LY294002 was from Cell Signaling Technology (Danvers, MA).

#### Antibodies

Antiphospho-Ser^473^-AKT (#4060), antiphospho-Thr^308^-AKT (#2965), total AKT (#4691), AKT1 (#2938), AKT2 (#2964), AKT3 (#8018), antiphospho-Ser^136^-BAD (#4366), antiphospho-Ser^9^-GSK3β (#5558), antiphospho-Thr^24^-FoxO1/-Thr^32^-FoxO3 (#9464), antiphospho-Ser^2448^-mTOR (#5536), antiphospho-Ser^2481^-mTOR (#2974), antiphospho-Thr^202^/Tyr^204^-p44/42 MAPK (Erk1/2) (#9101; #4370), total p44/42 MAPK (Erk1/2) (#9102) and cleaved caspase-3 (#9664) antibodies were purchased from Cell Signaling Technology (Danvers, MA); Anti-Beta Actin antibody (sc-1615) was purchased from Santa Cruz Biotechnology (Dallas, TX). Peroxidase-labeled affinity purified anti-rabbit IgG (074–1506), anti-mouse IgG (074–1806), anti-mouse IgM (074–1803) and anti-goat IgG (14-13-06) antibodies were purchased from KPL (Gaithersburg, MD).

### Methods

#### Whole Transcriptome Gene Expression Analysis

Clone9, N1S1 and AS30D cells were rinsed in 1xPBS, pelleted and snap frozen in liquid N_2_ (N = 4 biological replicates per cell line). Total RNA was extracted using RNeasy Mini Kit (Qiagen, Valencia, CA) per manufacturer protocol. RNA integrity was assessed using Agilent 2100 Bioanalyzer. All samples were processed using microarray technology with the Rat 230 2.0 Gene Chip (Affymetrix, Santa Clara, CA) and analyzed in the Mayo Clinic Medical Genome Facility (MCF) Gene Expression and Bioinformatics Cores. See S1 and S2 Files for the original gene expression data and S3 File for details on gene expression data analysis and Ingenuity pathway analysis.

#### Cross-comparison of gene expression data

Gene expression signatures from the three cell lines were compared to gene expression signatures from human liver and HCC (NCI cohort) using previously described methods [29, 33]. N1S1 and AS30D HCC molecular prognostic subtypes were classified based on the previously described poor prognostic hepatic stem cell (Cluster A and Subtype HS) or better prognostic hepatocyte (Cluster B and Subtype HCC) human HCC molecular prognostic subtypes [29].

#### Cell Proliferation and Metabolism

Cell proliferation was assessed using the MTT assay per manufacturer guidelines (ATCC, Manassas, VA). Doubling time and metabolic rate (mean±SD) were calculated from the absorbance data as previously described [36].

#### Cellular Heat Stress Protocol

The indicated cell lines were suspended in complete media in 1.5ml microcentrifuge tubes and heat stressed at the indicated temperatures in an isothermic water bath [24, 37]. Treatment temperature was monitored using an Omega HH41 digital thermometer (Omega Engineering, Stamford, CT) and maintained to within ±0.05°C. Adherent cell lines were prepared following dissociation using non-enzymatic cation-free HBSS/0.5% EDTA. Cells were pre-treated with the designated small molecule inhibitors or vehicle control ± heat stress as indicated. Cells were harvested at the indicated timepoints, rinsed in 1xPBS and snap frozen in liquid N_2_ for immunoblot analysis or analyzed with the WST-1 cell viability (Roche, Indianapolis, IN), CaspaseGlo® 3/7 apoptosis (Promega, Fitchburg, WI) or colony formation assay.

#### Cell Viability and Caspase 3/7 Activity

At the indicated times post heat stress, WST-1 assay (Roche, Indianapolis, IN) or CaspaseGlo® 3/7 assay (Promega, Fitchburg, WI) was performed per manufacturer instruction [37]. Absorbance (WST-1) or luminescence (CaspaseGlo®) was measured on a DTX 880 microplate reader (Beckman Coulter, Chaska, MN) and data were normalized to the non-heat stressed (37°C) vehicle control to determine relative cell viability and plotted versus temperature or Caspase 3/7 activity.

#### Soft Agar Colony Formation Assay (Clonogenic Survival)

The cell suspension was mixed with media (adherent cells) or sterile McCoy’s plus medium/agar solution (suspension cells), transferred to sterile tissue culture plates, allowed to set at room temperature for 20–30 minutes and placed in a humidified 37°C, 7.5% CO_2_ incubator for 7–14 days. Two to four technical replicates and three biological replicates were used per experimental condition. After 7–14 days, each dish was qualitatively assessed for evidence of colony formation and colonies of 50 or more cells were counted using a light microscope. Percent colony formation per cell number plated was calculated and the data normalized to the non-heat stressed, 37°C vehicle control to calculate percent colony formation.

#### Western Immunoblotting

Whole-cell lysates were separated by SDS-PAGE, electrophoretically transferred to PVDF and blotted for the indicated antigens using the indicated primary antibodies per manufacturer’s instruction followed by incubation with corresponding peroxidase labeled secondary antibodies (1:8000), incubated with enhanced chemiluminesence (ECL, Pierce, Thermo Fisher, Minneapolis, MN), exposed to film (Kodak/Carestream, Rochester, NY) and developed using a Kodak X-Omat M20 processor. Digital images were captured using a Kodak 440CF gel documentation system.

#### Kinomeview® Profiling

Per Cell Signaling Technology (CST; Danvers, MA) protocol, 30 μg of total protein was run in each lane for western blotting using a panel of motif antibodies with broad reactivity to serine, threonine and tyrosine phosphorylation and developed as previously described (see S3 File for a description of Kinomeview® antibodies and target consensus site) [38]. http://www.cellsignal.com/contents/proteomics-services-ptmscan-direct-services/kinomeview-services/proteomics-kinomeview

#### Tumor Ablation in Orthotopic N1S1 and AS30D HCC Models

All animal studies were carried out in strict accordance with the recommendations in the Guide for the Care and Use of Laboratory Animals of the National Institutes of Health. The protocol was approved by the Institutional Animal Care and Use Committee (IACUC) of the Mayo Clinic (Protocol Number: A31010). All surgery was performed under ketamine/xylazine or inhalation anesthesia; buprenorphine analgesia was administered pre-operatively and all efforts were made to minimize suffering. The N1S1 orthotopic HCC model was developed and tumor-bearing rats were imaged using non-contrast enhanced 3T magnetic resonance imaging (MRI; GE Healthcare, Milwaukee, WI) to measure tumor size and confirm tumor location as previously described [24, 39, 40]. Briefly, adult male Sprague-Dawley rats (400-500g) were randomized to percutaneous thermal ablation (N = 6) or sham ablation (N = 3). All ablation experiments were performed using an FDA-approved 980-nm laser generator (Visualase, Houston, TX) as previously described [24, 37, 39, 40]. Under ultrasound-guidance using an L8-18i transducer (logiq E9 Ultrasound, GE Healthcare, Milwaukee, WI), a bare 400μm core optical laser fiber with a 1.0cm diffusing tip was percutaneously inserted through a 22-gauge introducer sheath at the tumor margin and a 22-gauge needle with a 25-gauge wire thermocouple (Valleylab, Boulder, CO) was inserted 4-5mm from the laser fiber tip within the tumor for intraprocedural temperature monitoring. For the ablation group, tumors were ablated at a power setting of 3 watts (W) under continuous US-monitoring and the ablation stopped when the thermocouple reached 45°C in order to generate an intentional partial ablation. The laser was not turned on for sham-ablated animals. Immediately post-ablation rats underwent both non-contrast and gadolinium-enhanced 3T MRI to image post-ablation tissue changes as previously described [39]. Rats were euthanized at 6 or 24 hours post-ablation to assess early liver/tumor response to thermal injury. Experiments were repeated in the AS30D HCC model using methods previously described [41].

#### Gross and Microscopic Pathology

Liver/tumor tissue was removed and 2-mm cross-sections cut encompassing the ablation-zone. Vital staining with 0.5% triphenyltetrazolium chloride (TTC; Sigma, St. Louis, MO) was performed as previously described [42]. All specimens were formalin-fixed paraffin embedded (FFPE) and sectioned for histopathologic and immunohistochemical analysis. FFPE sections were stained with hematoxylin-eosin (H&E) or antiphospho-antibodies against the indicated antigens per manufacturer instruction as previously described [24, 37, 39, 41]. All sections were reviewed in a blinded and random fashion by an experienced pathologist (>20 years).

#### Human HCC AKT Gene Expression v. Recurrence and Tumor Pathologic Features

Oligonucleotide microarray analysis was performed on RNA from 139 pairs of HCC tumor and adjacent benign tissue from individuals who underwent surgical resection for primary HCC at centers in Asia, Europe and the United States as previously described [29, 32–35]. De-identified anonymized samples were either from patients who had provided verbal or written informed consent or were surgical waste tissues from deceased patients, which were used with IRB approval. The demographic, clinical, follow-up and pathological features for the 139 HCCs were previously summarized [29, 34]. The data have been deposited at Gene Expression Omnibus (GEO/GPL1528, GEO/GPL1529, GEO/GSE1897, GEO/GSE1898and GEO/GSE4024). Briefly, total RNAs were isolated from frozen liver tissue using CsCl density-gradient centrifugation. 20 mg of total RNA from tissues was used to derive fluorescently (Cy-5 or Cy-3) labeled cDNA. At least two hybridizations were performed for each tissue using a dye-swap strategy to eliminate dye-labeling bias. Specimens were analyzed at the US National Cancer Institute using the Human Array-Ready Oligo Set (Version 2.0) containing 70-mer probes of 21,329 genes from Qiagen (Valencia, CA). Expression ratios of each gene were averaged from duplicate experiments and data were plotted as a log2–fold change of AKT1, AKT2 and AKT3 mRNA expression, tumor over benign. Differences between the Kaplan-Meier curves of HCC patients with up-regulated or down-regulated AKT1, AKT2 and AKT3 expression in the HCC tissue in comparison with the adjacent benign tissue were analyzed with the log rank test. AKT1, AKT2 and AKT3 mRNA expression, tumor over benign, was compared by HCC prognostic cluster (A versus B), subtype (hepatocyte HC v. hepatoblast HS), tumor grade, vascular invasion and metastasis [29, 33].

#### Statistical Analyses

Statistical analyses were performed using Prism 5.0 (GraphPad Software, Inc., La Jolla, CA). Differences between treatment groups were compared with an unpaired *t* test (or Exact Mann-Whitney test) or one-way analysis of variance (ANOVA) followed by post-hoc pairwise comparison using an unpaired *t* test (or Exact Mann-Whitney test). Non-linear regression curve fitting was used to calculate a heat stress IT_50._ IT_50_ was defined as the temperature that induced a 50% reduction in cell viability relative to 37°C control for the indicated exposure time. See S3 File for details on cumulative equivalent minutes at 43°C (CEM43) calculations [43].

## Results

### Comparative genomic and biological characterization of Clone9 rat hepatocyte and N1S1 and AS30D rat HCC cell lines

#### Comparative Genomics

**Fig 1:**
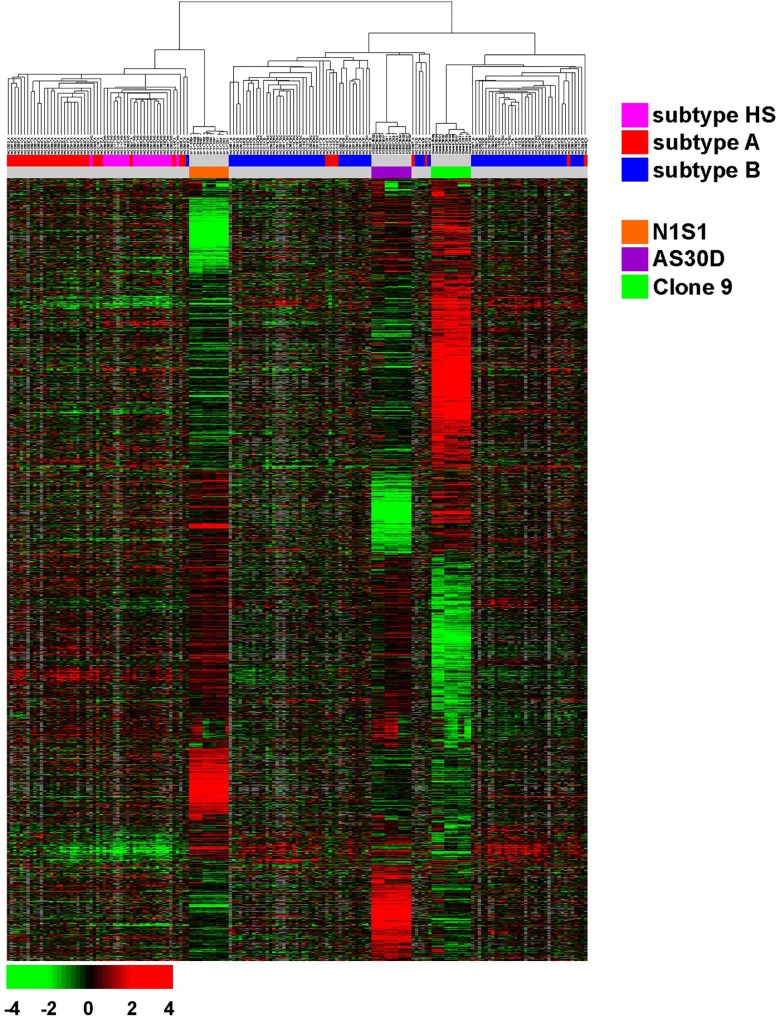
Cross comparison of integrated gene expression data from Clone9 rat hepatocyte and N1S1 and AS30D rat HCC cell lines with human HCC (NCI cohort). Hierarchical clustering analysis of integrated gene expression data from rat cell lines and human HCC tissues. Gene expression data from rat cell lines and human HCC tissues were independently centralized across samples to remove baseline differences between the two data sets. Genes with an expression level at least 2-fold different relative to the median value across tissues in at least 3 tissues in the rat data set were selected for hierarchical clustering analysis (2,776 unique genes). The data are presented in matrix format in which rows represent individual genes and columns represent each tissue. Each cell in the matrix represents the expression level of a gene feature in an individual tissue. The red and green color in cells reflect relative high and low expression levels respectively as indicated in the scale bar (log2 transformed scale). HS, A and B = prognostic subtypes.

Cross-comparison of the Clone9 rat hepatocyte and N1S1 and AS30D rat HCC cell line gene expression signature with orthologous human normal liver and HCC gene expression data demonstrated that the N1S1 cell line has a gene-expression signature consistent with a poor prognostic hepatic stem cell (Cluster A and Subtype HS) HCC molecular subtype while the AS30D cell line has a gene expression signature consistent with a better prognostic hepatocyte (Cluster B and Subtype HC) HCC molecular subtype (Fig 1). Moreover, the Clone9 rat hepatocyte cell line demonstrated a gene expression signature consistent with benign human liver (S1 Fig). The corresponding gene expression data for the N1S1 HCC, AS30D HCC and Clone 9 hepatocyte cell lines are available in S1 and S2 Files.

#### Cell Proliferation, Metabolic Rate and Colony Formation

Doubling time (hours) and metabolic rate (uU/hour/cell) are summarized in S1 Table. Doubling time of HCC cells relative to Clone9 hepatocytes was approximately 1.3 times faster for N1S1 (p<0.001) and 1.3 times slower for AS30D (p<0.01) while doubling time of N1S1 was 1.8 times faster than AS30D (p<0.0001). The metabolic rate of HCC cells relative to Clone9 hepatocytes was 2.4 times higher for N1S1 (p<0.0001) and 1.7 times higher for AS30D (p<0.001) while the metabolic rate of N1S1 was 1.4 times higher than AS30D (p<0.01). The N1S1 and AS30D rat HCC cell lines demonstrated evidence of colony formation in soft agar whereas the Clone9 rat hepatocyte cell line formed very few discrete, countable colonies (images not shown). Morphologically, the N1S1 colonies showed irregular borders and evidence of cell migration while the AS30D colonies showed regular borders and no evidence of cell migration (S2 Fig).

#### Ingenuity Pathway Analysis

The top biological functions, molecular and cellular functions, canonical pathways, transcription factors and up-regulated and down-regulated molecules based on comparison of differentially expressed genes for N1S1 HCC v. Clone9 hepatocyte and AS30D HCC v. Clone9 hepatocyte are summarized in S2–S5 Tables.

### Poor prognostic hepatic stem cell HCC subtype demonstrates enhanced survival to heat stress

**Fig 2:**
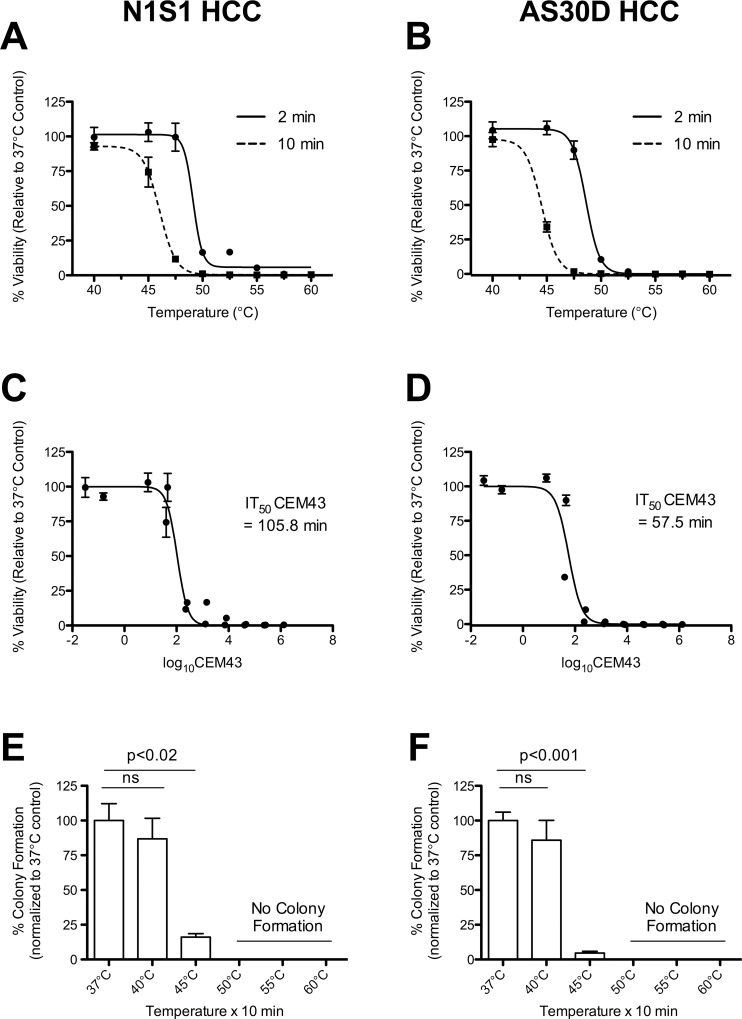
Effect of heat stress on N1S1 and AS30D cell viability and clonogenic survival. (A,B). Effect of heat stress temperature and exposure time on N1S1 and AS30D HCC cell viability. Cells heat-stressed at the indicated temperatures from 37°C-60°C for 2 (solid line) or 10 (dashed line) minutes were assessed with WST-1 viability assay at 72 hours post-heat stress. Data were normalized to 37°C control and presented as mean±SEM of 3 independent experiments. (C,D) IT_50_ for cumulative equivalent minutes at 43°C (CEM43) of N1S1 and AS30D HCC cells. N1S1 and AS30D heat stress temperature (°C) and exposure time (minutes) data 72-hours post heat stress were converted to CEM43 using equation (1), log_10_ transformed and plotted versus viability. An IT_50_ (95% confidence interval) for CEM43, heating time in minutes at 43°C to achieve a 50% kill, was calculated using non-linear regression. Data were normalized to 37°C control and presented as mean±SEM of 3 independent experiments. (E,F) Effect of heat stress on clonogenic survival of N1S1 and AS30D HCC cells. N1S1 and AS30D cells heat-stressed at the indicated temperatures from 37°C-60°C for 10 minutes were plated in soft-agar and colonies of 50 or more cells were counted 10 days after plating using a light microscope. Percent colony formation per cell number plated was calculated and the data normalized to the non-heat stressed, 37°C control to calculate percent colony formation relative control to 37°C control. Data are presented as mean±SEM of 3 independent experiments (One-way ANOVA followed by post-hoc pairwise comparison using an unpaired t-test).

To investigate the effects of temperature and exposure time (i.e. thermal dose) on the kinetics of heat stress induced cytotoxicity, N1S1 and AS30D HCC cell lines were heat stressed across the temperature range from physiologic (37°C) to complete cytotoxicity (60°C) for 2 or 10 minutes and assessed for cell viability at 6 to 72 hours post-heat stress [24]. The dose-response curves demonstrated a left shift for the 10-minute versus 2-minute exposure times for both cell lines (Fig 2A and 2B). The IT_50_ (in °C) for heat stress exposure times of 2 and 10 minutes are summarized in Table 1. For the 10 minute exposure time, the IT_50_ was significantly higher for the N1S1 compared to AS30D cells at 48 and 72 hours post heat stress (p<0.0001). After normalizing these heat stress exposure time and temperature data using the equation for continuous equivalent minutes at 43°C (CEM43), the heating time in minutes at 43°C to achieve a 50% kill (IT_50_ CEM43 [95% Confidence Interval]) was significantly longer for the N1S1 relative to the AS30D cell line (1.84 times longer; 105.8 minutes [84.6 to 132.5] v. 57.5 minutes [45.3 to 72.9], respectively) (Fig 2C and 2D). Taken together, the results of these experiments suggest that the poor prognostic HCC molecular subtype (N1S1) requires a higher thermal dose for cell killing than the better prognostic HCC molecular subtype (AS30D).

**Table 1:** IT_50_ (°C)^†^ for heat stress induced cytotoxicity in N1S1 and AS30D HCC cells.

**2min IT**_**50**_ **(°C)**	**6 Hour** *	**24 Hour** *	**48 Hour** *	**72 Hour** *
N1S1 HCC	>57.5	52.5 (51.3, 53.8)	49.3 (48.5, 50.0)	49.3 (48.3, 50.4)
AS30D HCC	>55.0	51.7 (50.7, 52.6)	48.9 (48.6, 49.2)	48.8 (48.5, 49.0)
**10min IT**_**50**_ **(°C)**	**6 Hour** *	**24 Hour** *	**48 Hour** *	**72 Hour** *
N1S1 HCC	53.1 (50.5, 55.8)	47.4 (46.1, 48.7)	45.7 (45.5, 45.8)	45.9 (45.6, 46.2)
AS30D HCC	51.8 (50.5, 53.0)	47.4 (46.6, 48.2)	44.4 (44.2, 44.6)	44.5 (44.3, 44.7)

Data are presented as IT_50_ (95% confidence interval), Comparison of 10 min IT_50_ at 48 and 72 hours between N1S1 and AS30D (p<0.0001)

* Indicates the time post heat stress the cells were assayed for viability.

^**†**^ IT_50_ is defined as the temperature that results in a 50% reduction in cell viability relative to 37°C control for the indicated exposure time.

Next, because both the N1S1 and AS30D HCC cell lines demonstrated different sensitivities to heat stress, we assessed the dose-dependent effects of heat stress on HCC clonogenic survival as an *in vitro* model for recurrence following heat stress. Heat stress at 40°C had no effect on colony formation relative to the untreated control (37°C) while heat stress at ≥50°C resulted in complete loss of colony formation in both cell lines (Fig 2E and 2F). Heat stress at 45°C resulted in a significant reduction in colony formation relative to the untreated control in both N1S1 and AS30D cell lines (p<0.02 and p<0.001, Fig 2E and 2F, respectively). However, the relative percent colony formation at 45°C was significantly greater in the N1S1 versus the AS30D cell line (3.5 fold; 16.1%±2.5% versus 4.7%±1.2%, respectively, p<0.02). These data identified experimental heat stress conditions which result in *in vitro* clonogenic survival in both cell lines (45°C for 10 minutes) and suggest that different molecular subtypes of HCC may have a different capacity to recover and progress following sublethal heat stress.

### Sublethal heat stress induces AKT and ERK survival signaling *in vitro* and thermal ablation induces AKT and ERK phosphorylation at the tumor ablation margin *in vivo*

#### Sublethal heat stress induces rapid changes in kinome-wide protein phosphorylation in HCC cells and hepatocytes

Preliminary experiments identified *in vitro* heat stress conditions (45°C for 10 minutes) that induce a significant reduction in cell survival but still result in colony formation (Fig 2E and 2F). This model recapitulates the thermal injury experienced at the margin of the ablation zone where both neoplastic and parenchymal cells experience heat stress that may or may not induce complete cell killing. Based on the kinetics of heat stress induced cytotoxicity, we hypothesized that sublethal heat stress induces rapid changes in cell signaling. Using this experimental model of post heat stress *in vitro* recurrence, we sought to examine global changes in protein phosphorylation and other post-translational modifications that are responsive to heat stress in order to identify candidate kinase families and pathways that may regulate HCC cell survival to heat stress. Immunoblot analysis identified significant changes in one or more bands for the AKT substrate motif, RXX(s/t) and tXR motif, tXR across all cell lines (S3 Fig). Modest changes in phosphorylation were observed for CK, PKA, PKC, PLC and Phospho-Thr-Pro motif antibodies (data not shown). These data demonstrate that heat stress induces rapid changes in the global phosphoproteome in both hepatocytes and HCC cells of rat and human origin.

#### Sublethal heat stress induces rapid AKT and ERK phosphorylation in vitro and thermal ablation induces AKT and ERK phosphorylation at the tumor ablation margin in vivo

Kinomeview® profiling showed that heat stress induces rapid changes in phosphorylation of AKT substrates containing the RXX(s/t) motif as well as phosphorylation of proteins containing the phosphor-Thr-X-Arg/Lys motif such as Raf (MAP3K), a serine/threonine kinase member of the mitogen-activated kinase family known to be phosphorylated at threonine followed by Lys at the +2 position (S3 Fig) [44]. Phosphorylated Raf then phosphorylates and activates MEK1/2 (MAP2K), a tyrosine/threonine kinase which in turn phosphorylates and activates ERK1/2 (MAPK3/MAPK1), a serine/threonine kinase [45]. Moreover, AKT is dually phosphorylated at Thr308 by PI3K signaling and Ser473 by mammalian target of rapamycin complex 2 (mTORC2) signaling while ERK1/2 is dually phosphorylated at Threonine202 and Tyrosine 204 by MEK for maximal activation [45–47]. The AKT and ERK pathways play critical roles in cell survival and proliferation and both are frequently dysregulated in numerous cancers, including HCC [45, 48–54].

**Fig 3:**
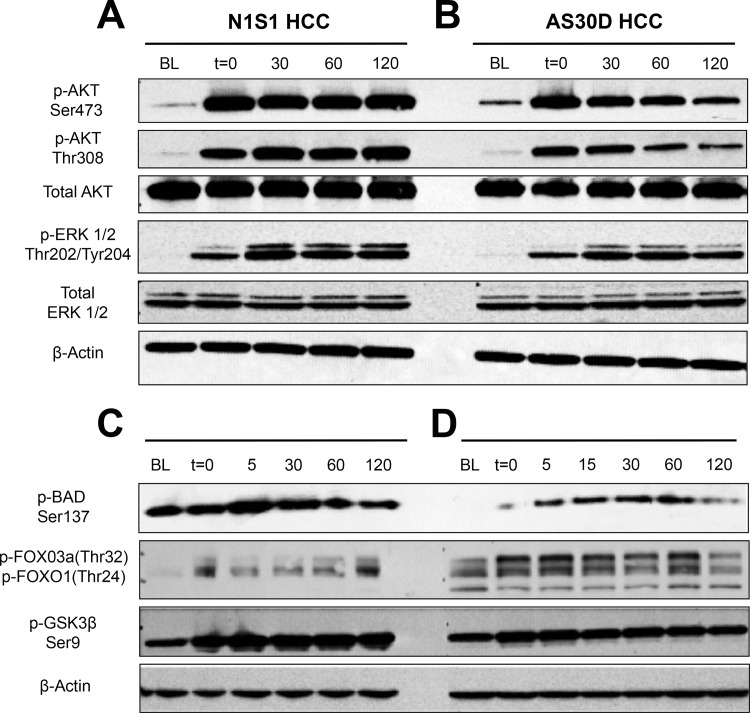
Effect of sublethal heat stress on AKT and ERK signaling in HCC cells. N1S1 and AS30D cells were heat stressed (45°C-10 minutes), recovered up to 2-hours post heat stress and whole-cell lysates were subjected to western immunoblotting for (A and B) phospho- and total AKT and ERK1/2, and (C and D) phospho-BAD, FOXO3a/FOXO1 and GSK3β. β-actin was used as a loading control. Representative images from 1 of 3 independent experiments. BL = baseline, non-heat stress control; t = time post heat stress. For example, t = 0 indicates immediate post-heat stress.

Given the results of the Kinomeview® analysis, we hypothesized that heat stress induces phosphorylation of AKT and ERK signaling. To test this hypothesis, N1S1 and AS30D cells were heat stressed and analyzed using phospho-specific antibodies against AKT and ERK by western immunoblotting (Fig 3A and 3B). Compared to baseline, heat stress induced a rapid increase in phosphorylation of both AKT and ERK immediately after the heat stress in both HCC cell lines. This increased phosphorylation of both AKT and ERK was sustained for at least 2 hours in the N1S1 cell line but began to decrease by 2 hours post heat stress in the AS30D cell line. Additionally, heat stress induced a time-dependent increase in the phosphorylation of AKT substrates 1) Bad, 2) FoxO3a/FoxO1, and 3) glycogen synthase kinase-3 (GSK3β), (Fig 3C and 3D). Phosphorylation of Bad, FoxO3a/FoxO1 and GSK3β by AKT negatively regulates their activity, thereby promoting cell survival [55–57]. Finally, similar experiments confirmed that heat stress induced a rapid, differential increase in the phosphorylation of AKT, GSK3β and ERK in a panel of eight human HCC cell lines (S4 Fig).

**Fig 4:**
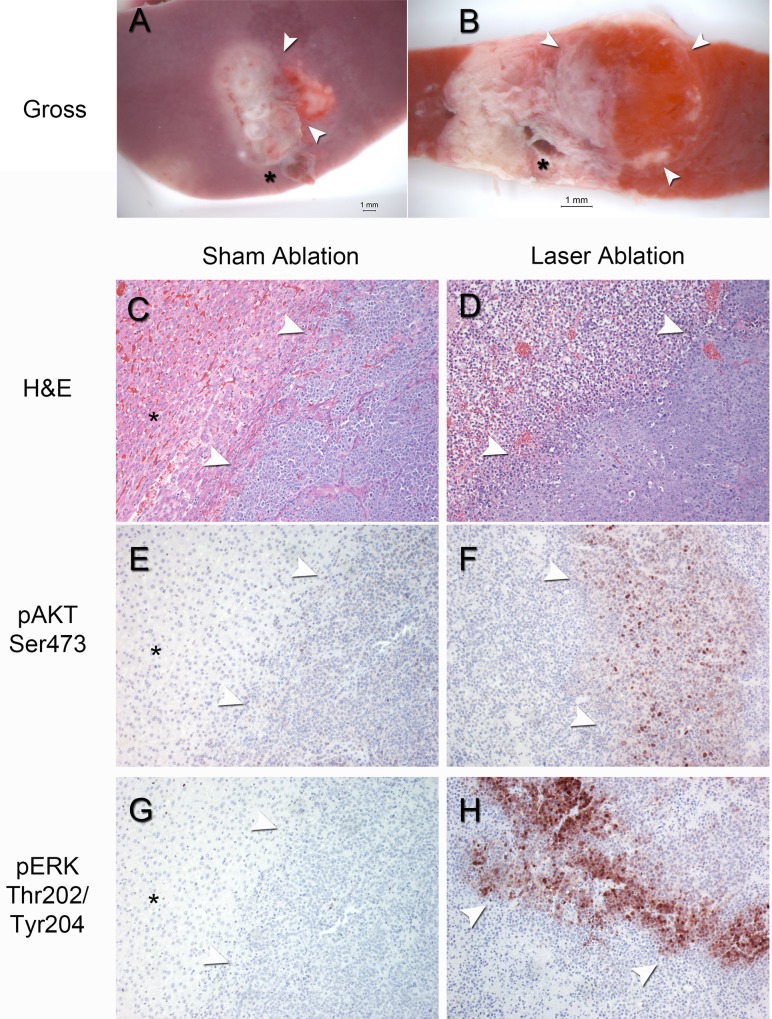
Representative gross and microscopic pathology and immunohistochemical staining of the ablation zone 24-hours post-ablation in the orthotopic N1S1 HCC model. (A-B) A) Ablation zone *in situ* demonstrates a whitish-appearing N1S1 tumor within the liver and a hyperemic rim at the edge of the ablation zone (denoted by white arrowheads). B) Triphenyltetrazolium chloride (TTC) vital staining of cross-section through the ablation zone containing both liver and tumor demonstrates N1S1 tumor within the liver (denoted by white arrowheads) and areas of viable (red) and non-viable (white) tumor and liver within the ablation zone consistent with thermal-ablation induced tissue coagulation. * = laser fiber tract. (C-H) Representative histopathology and phospho-AKT and ERK immunostaining of N1S1 tumors from sham and laser ablation groups. Photomicrographs (100x) of H&E stained sections (C, D) demonstrate C) the liver-tumor margin of a sham-ablated tumor (denoted by white arrowheads) and the D) tumor-ablation margin of a laser ablated tumor (denoted by white arrowheads). Corresponding photomicrographs (100x) of p-AKT (E,F) and p-ERK (G,H) immunostained sections demonstrate markedly increased AKT (F) and ERK (H) phosphorylation at the tumor-ablation margin (denoted by white arrowheads) in the laser-ablated tumor but minimal AKT (E) and ERK (G) phosphorylation in the tumor, background liver or at the tumor-liver margin (denoted by white arrowheads) in the sham-ablated tumor. (*) denotes background liver.

Having identified that heat stress activates both AKT and ERK signaling in a range of HCC cell lines, we sought to confirm whether these pathways are induced by thermal ablation *in vivo* (S5 and S6 Figs). Gross analysis of the ablation zone *in situ* (Fig 4A) and in cross-section with TTC vital staining demarcated the ablation margin and boundaries between viable (red) and non-viable (white) tumor and liver tissue within the ablation zone (Fig 4B). H&E and immunohistochemical analysis of the ablation zone after laser ablation demonstrated discrete morphologic regions within the ablation zone that correlated with TTC vital staining (Fig 4D) and markedly increased immunostaining for phospho-AKT (Fig 4F) and phospho-ERK (Fig 4H) at the tumor ablation margin but not at distances further from the tumor ablation margin or at the liver ablation margin (S7 Fig for phospho-AKT). On the other hand, there was no evidence of increased immunostaining for phospho-AKT (Fig 4E) and phospho-ERK (Fig 4G) in the tumor or at the margin between liver and tumor in the sham ablation group. In short, these data suggest that thermal ablation induces AKT and ERK phosphorylation at the tumor ablation margin *in vivo*. Similar results were obtained with the AS30D orthotopic HCC model (S8 Fig).

### Sublethal heat stress induces PI3K/mTOR and MEK-dependent AKT and ERK signaling, respectively, and inhibition of the PI3K-AKT-mTOR pathway but not MEK enhances heat stress induced HCC cell killing

**Fig 5:**
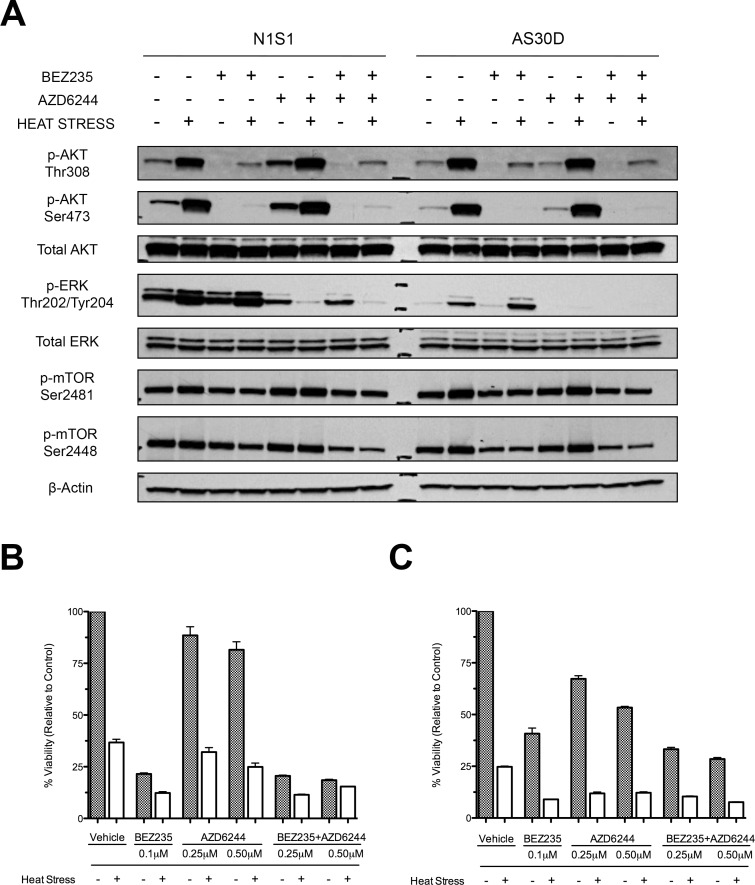
Effect of PI3K/mTOR and/or MEK inhibition on heat stress induced AKT, mTOR and ERK signaling and cytotoxicity in HCC cells. (A) N1S1 and AS30D cells were pre-treated for one-hour with NVP-BEZ235 (0.5μM), AZD6244 (1.0 μM), both or vehicle control (0.1% DMSO) followed by heat stress (45°C) or control (37°C) for 10 minutes. Immediately post-heat stress whole-cell lysates were subjected to western immunoblotting for phospho- and total AKT and ERK, and phospho-mTOR. β-actin was used as a loading control. Representative images from 1 of 3 independent experiments (B) Effect of PI3K/mTOR and/or MEK inhibition on heat stress induced cytotoxicity in HCC cells. N1S1 and AS30D cells pre-treated for one-hour with NVP-BEZ235 (0.1μM), AZD6244 (0.25 or 0.5 μM), both or vehicle control (0.1% DMSO) followed by heat stress (45°C) or control (37°C) for 10 minutes were assessed using the WST-1 viability assay at 48 hours post-heat stress. Data were normalized to 37°C vehicle control and presented as mean±SEM of 3 independent experiments.

Next we sought to investigate the upstream mechanisms mediating heat stress induced AKT and ERK signaling. Western immunoblotting demonstrated that inhibition of PI3K/mTOR with NVP-BEZ235 and MEK with AZD6244 blocked constitutive and heat stress induced AKT and ERK phosphorylation, respectively (Fig 5A) [58, 59]. Additionally, heat stress induced autophosphorylation of mTOR at Ser2481, a marker for activation of mTORC2 but did not induce phosphorylation of mTOR at Ser2448, a marker for mTORC1 signaling [60]. Inhibition of PI3K/mTOR blocked both mTORC1 and mTORC2 kinase signaling. Lastly, MEK inhibition alone increased basal AKT phosphorylation in the N1S1 cell line but not the AS30D cell line, suggesting differential cross-talk between PI3K-AKT-mTOR and RAS-RAF-MEK-ERK signaling between these two cell lines. Viability assessment confirmed that dual PI3K/mTOR inhibition has strong cytotoxic effects as a single agent and enhances heat stress induced HCC cell killing in both cell lines (Fig 5B and 5C). MEK inhibition induced modest, dose-dependent cytotoxic effects as a single agent in the AS30D cell line (Fig 5C) but an IT_50_ was not achieved in the N1S1 cell line, even at the 500nM dose (Fig 5B). Moreover, inhibition of MEK did not enhance heat stress induced HCC cell killing in the N1S1 cell line and only moderately increased heat stress induced cell killing in the AS30D cell line. Finally, combination PI3K/mTOR and MEK inhibition did not further enhance heat stress induced cell killing over PI3K/mTOR inhibition alone in either cell line [61]. Given these findings, the PI3K-AKT-mTOR pathway was chosen for further investigation.

**Fig 6:**
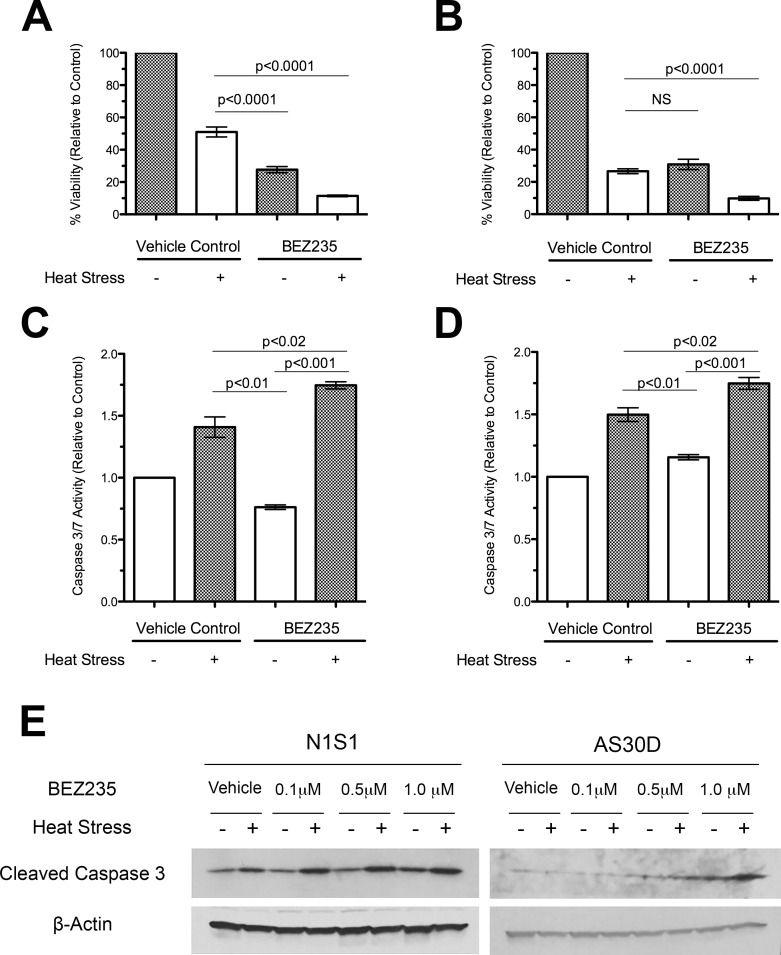
Effect of PI3K/mTOR inhibition on heat stress induced cytotoxicity and apoptosis in HCC cells. (A-B) Effect of NVP-BEZ235 on heat stress induced cytotoxicity in HCC cells. (A) N1S1 and (B) AS30D cells pre-treated for one hour with NVP-BEZ235 (0.1 μM) or vehicle control (0.1% DMSO) followed by heat stress (45°C) or control (37°C) for 10 minutes were assessed with WST-1 viability assay at 72 hours post-heat stress. Data were normalized to 37°C vehicle control and presented as mean±SEM of 3 independent experiments. (One-way ANOVA followed by post-hoc pairwise comparison using an unpaired t-test). (C-D) Effect of PI3K/mTOR inhibition on heat stress induced caspase 3/7 activity in HCC cells. (C) N1S1 and (D) AS30D cells pre-treated for one hour with NVP-BEZ235 (0.1 μM) or vehicle control (0.1% DMSO) followed by heat stress (45°C) or control (37°C) for 10 minutes were assessed with the CaspaseGlo® 3/7 assay at 48 hours post-heat stress. Data were normalized to 37°C control and presented as mean±SD (N = 4) (One-way ANOVA followed by post-hoc pairwise comparison using an unpaired t-test). (E) Effect of PI3K/mTOR inhibition on generation of cleaved caspase 3 in HCC cells. N1S1 and AS30D cells were pre-treated for one hour with the indicated dose of BEZ235 or vehicle control (0.1% DMSO) followed by heat stress (45°C) or control (37°C) for 10 minutes. At 24 hours post-heat stress whole-cell lysates were subjected to western immunoblotting for cleaved caspase 3. β-actin was used as a loading control. Representative images from 1 of 2 independent experiments

Western immunoblotting demonstrated that both NVP-BEZ235 and LY294002 inhibitors prevented both constitutive and heat stress induced AKT phosphorylation at Thr308 and Ser473 in both cell lines (S9A Fig) [62]. Viability assessment demonstrated that both PI3K and dual PI3K/mTOR inhibition increased heat stress-induced HCC cell killing over heat stress or inhibitor alone (p<0.001; S9A and S9B Fig). Moreover, the dual PI3K/mTOR inhibitor NVP-BEZ235 was as efficacious (N1S1; S9B Fig) if not more (AS30D; S9C Fig) than the PI3K inhibitor LY294002. Based on the potency and clinical relevance of the dual PI3K/mTOR inhibitor NVP-BEZ235, this drug was chosen for subsequent experiments. Further experiments confirmed that inhibition of PI3K/mTOR enhances heat stress induced HCC cell killing over heat stress or drug alone (p<0.0001) (Fig 6A and 6B). Additionally, PI3K/mTOR inhibition in combination with heat stress increased caspase 3/7 activity (p<0.02; Fig 6C and 6D) and generation of cleaved caspase 3 in a dose-dependent manner (Fig 6E).

### PI3K/mTORC2-dependent AKT signaling is a central mediator of HCC cell survival to heat stress

The observation that sublethal heat stress induces rapid PI3K/mTOR dependent AKT signaling and that inhibition of PI3K/mTOR-dependent AKT signaling enhances heat stress induced HCC cell killing raises the question whether blockade of the entire PI3K-AKT-mTOR pathway vs. AKT alone mediates the increased HCC cell killing to heat stress. As such, we sought to determine which components of the PI3K-AKT-mTOR pathway mediate HCC cell survival to heat stress using a clonogenic survival assay as an *in vitro* model of recurrence (Fig 2E and 2F; S2 Fig).

**Fig 7:**
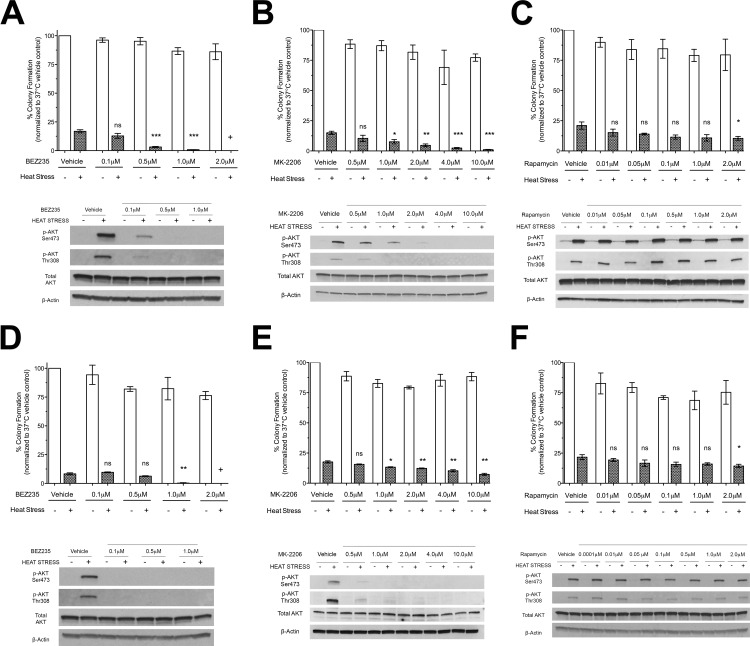
Effect of PI3K/mTORC1/2, AKT and mTORC1 inhibition on heat stress AKT signaling and clonogenic survival in HCC cells. (A-C) N1S1 and (D-F) AS30D HCC cells pre-treated with a dose-titration of (A,D) the dual PI3K/mTORC1/2 inhibitor NVP-BEZ235, (B,E) the AKT inhibitor MK-2206 (C, F) the mTORC1 inhibitor rapamycin or vehicle control (0.1% DMSO) followed by heat stress (45°C) or control (37°C) for 10 minutes were harvested immediately post-heat stress for western immunoblotting or plated for the colony formation assay. For immunoblotting, whole-cell lysates were subjected to western immunoblotting for phospho- and total AKT. β-actin was used as a loading control. Representative images from 1 of 3 independent experiments are shown. For the colony formation assay, colonies of 50 or more cells were counted 7 (N1S1) or 10 (AS30D) days after plating using a light microscope. Percent colony formation per cell number plated was calculated and the data normalized to the non-heat stressed, 37°C control to calculate percent colony formation relative control to 37°C control. Data are presented as mean±SEM of 3 independent experiments (One-way ANOVA followed by post-hoc pairwise comparison using an unpaired t-test). ns P > 0.05; * P ≤ 0.05; ** P ≤ 0.01; *** P ≤ 0.001; **** P ≤ 0.0001

Inhibition of both PI3K/mTORC1/2 (NVP-BEZ235) (Fig 7A and 7D) and AKT (MK-2206) (Fig 7B and 7E) blocked heat stress induced phosphorylation of AKT at Ser473 and Thr308 and significantly decreased HCC clonogenic survival to sublethal heat stress [58, 63]. In fact, inhibition PI3K/mTORC1/2 in combination with sublethal heat stress resulted in no colony formation at higher doses of NVP-BEZ235. Similar findings were obtained in the human HCC cell line Hep3B (S10A and S10B Fig). However, preferential inhibition of mTORC1 (Rapamycin) did not block heat stress induced AKT activation at Ser473 or Thr308 and had minimal effect on reducing HCC cell survival to sublethal heat stress, even at higher doses (Fig 7C and 7F) [64]. Of note, mTORC1/2 and mTORC1 inhibition alone significantly reduced Hep3B clonogenic survival suggesting that this HCC cell line may in part be mTOR-dependent at baseline (S10C Fig).

### AKT isoforms are differentially overexpressed in human HCC and correlate with worse HCC tumor characteristics and prognosis

**Fig 8:**
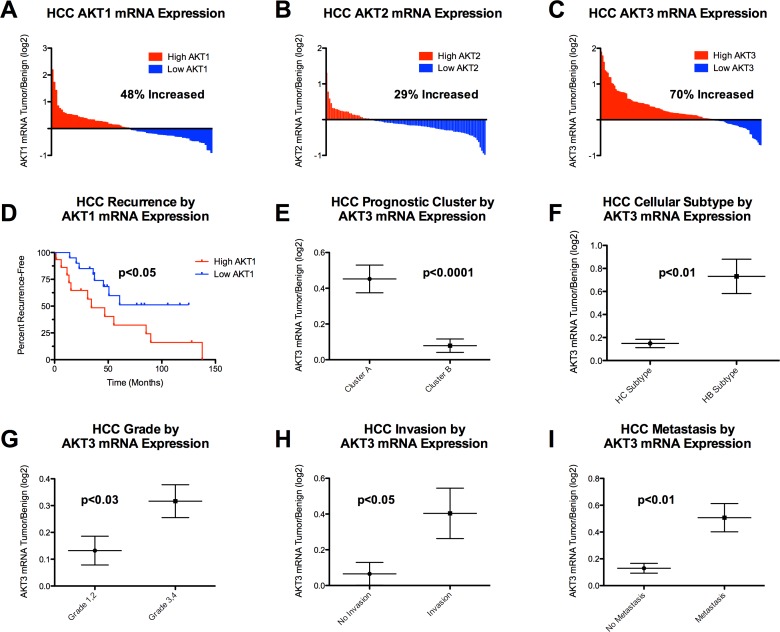
AKT isoforms are differentially upregulated in human HCC. AKT1 correlates with worse prognosis after surgical resection while AKT3 correlates with worse HCC tumor characteristics. (A-C) Results of oligonucleotide microarray analysis performed on 139 pairs of tumor and benign samples from primary HCCs, plotted as a log 2–fold change of AKT1, AKT2, AKT3 mRNA expression, tumor over benign. Red = increased expression and blue = decreased expression in tumor relative to benign adjacent tissue. (D) Kaplan-Meier survival analysis after surgical resection in the 20 patients with the highest tumor/benign AKT1 mRNA expression and in the 20 patients with the lowest tumor/benign AKT1 mRNA expression. Increased AKT1 is associated with more rapid recurrence after surgical resection of the primary tumor. (E-G) Correlation of HCC tumor characteristics with AKT3 expression. HCC tumors with (E) a poor prognostic molecular signature—cluster A, (F) hepatic stem cell (HS) subtype, (G) higher tumor grade, (H) vascular invasion and (I) metastasis had significantly greater AKT3 mRNA expression in tumor compared to benign adjacent tissue.

Given that AKT signaling may be an important mediator of HCC cell survival to thermal ablation induced heat stress, we sought to investigate the potential clinical significance of the three AKT isoforms in human HCC. There was increased expression in the HCC tumor compared to the benign adjacent tissue in the following proportions of HCCs: AKT1 (48.2%; Fig 8A), AKT2 (29.1%; Fig 8B) and AKT3 (70.0%; Fig 8C). Recurrence analysis showed that patients with tumors expressing the highest versus lowest levels of AKT1 had a more rapid rate of tumor recurrence after surgical resection (P<0.05; Fig 8D). AKT2 and AKT3 expression were not associated with more rapid recurrence (data not shown). Moreover, HCC tumors with a poor prognostic cluster A (p<0.0001; Fig 8E), hepatic stem cell (HS) subtype (p<0.01; Fig 8F), higher tumor grade (p<0.03; Fig 8G), vascular invasion (p<0.05; Fig 8H) and metastasis (p<0.01; Fig 8I) had significantly greater expression of AKT3. Given these findings we examined AKT isoform protein expression in our *in vitro* HCC model system (Fig 1). Western immunoblotting showed that all four cell lines express AKT1 and minimal AKT2 (S11 Fig). Interestingly, the N1S1 cell line, which has a poor prognostic hepatic stem cell molecular subtype of HCC (Cluster A and Subtype HS; Fig 1), also expresses high levels of AKT3, findings consistent with the human HCC gene expression data (Fig 8E and 8F). On the other hand, the AS30D cell line, which subclassifies with the better prognostic hepatocyte molecular subtype of HCC (Cluster B and Subtype HC) and the human Hep3B and HuH7 HCC cell lines do not express AKT3. In summary, these data show that AKT isoforms are differentially overexpressed in human HCC tumors and may have biological (AKT3) and prognostic significance (AKT1) in an isoform-dependent manner.

## Discussion

The clinical use of thermal ablation for HCC has outpaced the understanding of the basic molecular mechanisms mediating HCC-specific response to heat stress, particularly at the ablation margin where tumor recurrence remains a challenging problem. The experiments herein sought to address the following basic and translational questions regarding mechanisms of HCC cell survival to thermal ablation induced heat stress using a combination of *in vitro*, *in vivo* and patient based approaches: 1) What is the biological and clinical relevance of the N1S1 and AS30D HCC cell lines as models for testing thermal ablation hypotheses? 2) What signaling pathway(s) mediate HCC cell survival to sublethal heat stress? 3) Does inhibition of these pathway(s) in combination with heat stress decrease HCC cell survival? 4) Do these signaling pathways have biologic or prognostic significance to human HCC?

Previous studies have demonstrated that the N1S1 and AS30D HCC cell lines are tumorigenic when implanted in the liver of immunecompetent rats, are phenotypically distinct and are feasible models for testing thermal ablation hypotheses *in vivo* [24, 37, 39–41]. However, the biologic and clinical relevance of these cell lines has not been characterized, and the generalizability of this model system to human liver and HCC has remained unknown. As such, we sought to characterize the biological properties and clinical relevance of this *in vitro* model system. The comparative genomic data showed that the gene expression signature of the Clone 9 normal rat hepatocyte cell line is consistent with benign human liver while the gene expression signature of the N1S1 HCC cell line corresponds to a poor prognostic hepatic stem cell molecular subtype (Cluster A and Subtype HS) and the AS30D HCC cell line corresponds to a better prognostic hepatocyte (Cluster B and Subtype HC) of human HCC [29]. These three cell lines have several advantages as a candidate *in vitro* model system for examining molecular mechanisms mediating the variation in cellular response to heat stress between hepatocytes and different sub-types of HCC: 1) they are derived from the same cell type—hepatocyte—from the same species of rat; 2) they are phenotypically and biologically distinct representing two ends of the HCC disease spectrum and 3) the HCC cell lines are tumorigenic which facilitates translation from *in vitro* to *in vivo* thermal ablation studies (S6 Table for cell line comparison) [29].

Having established the biological and prognostic significance of the N1S1 and AS30D HCC cell lines, we sought to investigate the thermal-dose dependent effects of heat stress on HCC cytotoxicity using clinically relevant experimental heat stress conditions as well as the impact of HCC biological heterogeneity on sensitivity to heat stress induced cell killing *in vitro*. The *in vitro* findings demonstrate that the poor prognostic hepatic stem cell subtype of HCC (N1S1) requires a higher thermal dose for cell killing and has a greater capacity to recover and progress following sublethal heat stress than the better prognostic hepatocyte subtype of HCC (AS30D). Importantly, these studies identified experimental heat stress conditions (45°C for 10 minutes) that result in significantly different survival between HCC subtypes using experimental heat stress conditions relevant to the clinical thermal ablation zone [43, 65, 66]. These experimental conditions, defined as sublethal heat stress, were chosen to investigate the molecular mechanisms mediating the variation in response to sublethal heat stress between these two cell lines. Taken together, these data suggest that the molecular subtype of HCC may play a fundamental role in variation in HCC response to heat stress, particularly at lower thermal doses.

Next we sought to identify candidate signaling pathways that may mediate HCC cell survival to sublethal heat stress. The findings from the initial kinome screen suggested that both AKT and ERK signaling pathways may be induced by sublethal heat stress. Both the AKT and ERK pathways are known to play critical roles in cell growth, survival and proliferation and are frequently dysregulated in numerous cancers including HCC and predict a poorer prognosis [45, 48–54]. Subsequent experiments confirmed that sublethal heat stress induces rapid AKT and ERK phosphorylation in both rat and human HCC cells *in vitro*, findings consistent with a previous study [67]. However, these data build on previous studies by identifying important differences in the kinetics of heat stress induced AKT and ERK phosphorylation depending on the molecular subtype of HCC under clinically relevant heat stress conditions. Additionally, sublethal heat stress induced a time-dependent increase in the phosphorylation of AKT substrates Bad, FoxO3a/FoxO1 and glycogen synthase kinase-3 (GSK3β) whose phosphorylation negatively regulates their pro-cell death functions, thereby promoting a survival phenotype [55–57]. The *in vivo* ablation experiments confirmed that thermal ablation induces AKT and ERK phosphorylation at the HCC tumor ablation margin *in vivo*, the region of the ablation zone at highest risk for tumor recurrence. Given the known pro-survival effects of AKT and ERK signaling, these pathways were chosen as candidate mediators of HCC cell survival to sublethal heat stress for further investigation.

Next, we sought to determine if inhibition of AKT and ERK pathways in combination with heat stress enhances heat stress induced HCC cell killing. Experiments confirmed that inhibition of heat stress induced AKT signaling with the dual PI3K/mTOR inhibitor NVP-BEZ235 increased heat stress induced HCC cell killing. Conversely, inhibition of ERK signaling with the MEK inhibitor did not enhance heat stress induced HCC cell killing in the N1S1 cell line and only modestly increased heat stress induced cell killing in the AS30D cell line. The difference in the efficacy of MEK inhibition between these two cell lines may in part be explained by the paradoxical activation of AKT signaling upon treatment with the MEK inhibitor in the N1S1 cell line but not in the AS30D cell. As such, these findings would support the hypothesis that activation of AKT signaling may be a potential mechanism of resistance to treatment with inhibitors of RAS-RAF-MEK-ERK signaling in certain sub-types of HCC. Lastly, dual inhibition of heat stress induced AKT and ERK signaling did not further enhance heat stress induced HCC cell killing over AKT (PI3K/mTOR) inhibition alone [61]. Additional experiments further confirmed that inhibition of heat stress induced AKT signaling via PI3K or PI3K/mTOR inhibition significantly enhanced heat stress induced HCC cell killing, caspase 3/7 activity and cleaved caspase 3 generation, suggesting that inhibition of PI3K/mTOR may enhance heat stress induced cell killing by in part increasing apoptosis.

Next we sought to determine which components of the PI3K-AKT-mTOR pathway including PI3K/mTORC1/2, mTORC1 and/or AKT mediate HCC cell survival to heat stress using a clonogenic survival assay as an *in vitro* model of recurrence. These experiments provided further evidence that blocking functional activation of AKT via PI3K/mTORC2-dependent AKT phosphorylation or directly blocking AKT function significantly decreased HCC survival to heat stress while mTORC1 inhibition had no impact. Dual PI3K/mTORC2 inhibition with NVP-BEZ235 was the most efficacious and potent enhancer of heat stress induced HCC cell killing. These findings are consistent with previous studies demonstrating that dual PI3K/mTOR inhibitors enhance antitumor activity when used in combination with radiation, cytotoxic chemotherapy and molecularly targeted agents [58, 68–70]. In addition, it is known that inhibition of mTORC1 signaling alone using rapamycin may potentiate PI3K activation through inhibition of the mTORC1/S6K1 negative feedback loop. This may in part explain the lack of efficacy of rapamycin. Inhibition of both PI3K and mTOR kinase activity is now recognized as more effective at inhibiting AKT phosphorylation and activation [71]. Taken together, these data provide evidence that PI3K/mTORC2-dependent AKT signaling is a central mediator of HCC cell survival to heat stress and that the increased HCC cell killing is independent of mTORC1 signaling.

Finally, given that these data support that AKT signaling is an important mediator of HCC cell survival to thermal ablation induced heat stress, we sought to investigate the potential clinical significance of the three AKT isoforms in human HCC. Prior studies have shown that AKT isoform 1, 2 and 3 have diverse roles in mediating tumorigenesis and cancer cell survival [72–75]. Increased expression of AKT1, but not AKT3 or AKT2, predicted more rapid recurrence following surgical resection of HCC. Additionally, patients with tumors that had a poor prognostic hepatic stem cell subtype, vascular invasion and metastasis showed increased expression of AKT3 expression. Of note, the N1S1 HCC cell line expressed high levels of AKT3 whereas the other cell lines did not, thereby providing further evidence for the clinical relevance of the N1S1 HCC cell line as a worse prognostic subtype of human HCC. One hypothesis is that gain of AKT3 expression is associated with worsening tumor biology and phenotypic de-differentiation. Overall, these data provide further molecular insight into how AKT isoforms are differentially overexpressed in human HCC tumors and may have biological (AKT3) and prognostic significance (AKT1) in an isoform-dependent manner.

There are limitations to these studies. First, the genomic data and biological pathway analysis do not provide specific mechanistic insight into the regulation of HCC oncogenesis in the N1S1 and AS30D rat HCC cell lines. Rather, these data are hypothesis generating and provide a framework for understanding the biological and translational relevance of this model system to human HCC. Second, pharmacologic inhibition methods with small molecule kinase inhibitors were used to investigate heat stress induced PI3K-AKT-mTOR signaling. Although these are well-characterized, specific kinase inhibitors, they are prone to off target effects, particularly at higher doses. As such, multiple structurally dissimilar inhibitors with a dose-titration were used to examine the dose-dependent effects. Nonetheless, future gain and loss of function experiments will help to further delineate which component(s) of the PI3K-AKT-mTOR pathway and/or which AKT isoforms mediate HCC cell survival to heat stress. Moreover, the *in vitro* findings warrant further validation in clinically and biologically relevant HCC animal models using both pharmacologic and genetic methods of AKT inhibition. Third, while these experiments focused on heat stress induced AKT and ERK signaling as candidate mediators of HCC cell survival to heat stress, other survival pathways such as p38 MAPK, SAP/JNK and TGF-β signaling warrant further investigation.

## Conclusions

In summary, these findings support the hypothesis that heat stress induced, PI3K/mTORC2-dependent AKT signaling is a central mediator of HCC cell survival to heat stress and provide a mechanistic rationale for adjuvant PI3K-AKT-mTOR inhibition in combination with thermal ablation. Moreover, the N1S1 and AS30D HCC cell lines may serve as a relevant model not only of two biologically diverse molecular prognostic subtypes of HCC but also for investigating the isoform specific role of AKT in HCC tumorigenesis and survival to thermal ablation induced heat stress.

## Supporting Information

S1 Fig Cross comparison of integrated gene expression data from Clone9 rat hepatocyte and N1S1 and AS30D rat HCC cell lines with human benign liver and HCC (NCI cohort). Hierarchical clustering analysis of integrated gene expression data from rat cell lines and human HCC tissues. Gene expression data from rat cell lines and human HCC tissues were independently centralized across samples to remove baseline differences between the two data sets. Genes with an expression level at least 2-fold different relative to the median value across tissues in at least 3 tissues in the rat data set were selected for hierarchical clustering analysis (2,759 unique gene). The data are presented in matrix format in which rows represent individual gene and columns represent each tissue. Each cell in the matrix represents the expression level of a gene feature in an individual tissue. The red and green color in cells reflect relative high and low expression levels respectively as indicated in the scale bar (log2 transformed scale). ST = BL = Benign Liver. HCC = Hepatocellular Carcinoma.
(TIFF)

S2 Fig Soft-agar colony formation of N1S1 and AS30D rat HCC cell lines. Representative photomicrographs of N1S1 (A,C) and AS30D (B,D) colonies after 10 days at 100x (A,B) and 200x (C,D) magnification.
(TIF)

S3 Fig Immunoblot analysis of kinome-wide changes in protein phosphorylation in response to heat stress in hepatocytes and HCC cells. (A) Phospho-AKT Substrate RXX(S*T*) immunoblot; (B) Phospho-Thr-X-Arg Motif T*X(K/R). (C) β-Actin (top) and Gel Stain (bottom) control immunoblots. Odd numbered lanes are without heat stress and even numbered lanes are with heat stress. Lanes 1,2 = Clone rat hepatocyte; Lanes 3,4 = N1S1 rat HCC; Lanes 5,6 = AS30D rat HCC; Lane 7,8 = HuH7 human HCC; Lanes 9,10 = Hep3B human HCC; and Lanes 11,12 = PLC/PRF/5 human HCC.
(TIF)

S4 Fig Effect of sublethal heat stress on AKT and ERK signaling in human HCC cells. The indicated cell lines were heat stressed (45°C) or control (37°C) for 10 minutes, harvested immediately post-heat stress and whole-cell lysates were subjected to western immunoblotting using phospho-specific antibodies against AKT, GSK3β and ERK. β-actin was used as a loading control.
(TIF)

S5 Fig Percutaneous US-guided laser ablation with temperature monitoring in orthotopic N1S1 HCC model. A) Percutaneous insertion of laser fiber (*) and thermocouple (**) into N1S1 tumor under US guidance. B) Short axis and C) long axis US images demonstrate a hypoechoic tumor (white arrowheads) with the hyperechoic-appearing laser fiber and thermocouple located in the inferior-lateral and superior-medial aspects of the tumor, respectively.
(TIF)

S6 Fig Representative pre- and post-ablation non-contrast and gadolinium-enhanced axial fast spin echo (FSE) T2- and T1-weighted MR images of orthotopic N1S1 HCC model. A) Pre-ablation and B-F) post-ablation 3T MR images demonstrate A) a hyperintense T2-weighted N1S1 tumor pre-ablation (denoted by white arrowhead), B) decreased T2-signal and C) decreased T1-signal relative to liver on immediate post-ablation non-contrast enhanced MRI. Gadolinium-enhanced T1-weighted MR images at D) 3-minutes E) 6-minutes and F) 10 minutes post-injection demonstrate time-dependent enhancement of the background liver and a hypoenhancing zone in the region of the tumor ablation.
(TIF)

S7 Fig Representative phospho-AKT immunostaining of the tumor ablation and liver ablation margins in a laser-ablated orthotopic N1S1 tumor. A) low power (25x) and C) higher power (50x) photomicrographs demonstrate very few cells staining positive (brown) for phospho-AKT in the background liver or at the liver-ablation margin (denoted by black arrowheads). B) low power (25x) and D) higher power (50x) photomicrographs demonstrate focal areas of markedly increased phospho-AKT immunostaining at the tumor ablation margin (denoted by black arrowheads) with decreased immunostaining further from the ablation margin toward the non-ablated tumor.
(TIF)

S8 Fig Representative gross and microscopic pathology and immunohistochemical staining of the ablation zone 24-hours post-ablation in the orthotopic AS30D HCC model. Photomicrographs (100x) of H&E stained sections (A,B) demonstrate A) the liver-tumor margin of a sham-ablated tumor (denoted by white arrowhead) and the B) tumor-ablation margin of a laser ablated tumor (denoted by white arrowheads). Corresponding photomicrographs (100x) of p-AKT (C,D) and p-ERK (E,F) immunostained sections demonstrate markedly increased AKT (D) and minimally increased ERK (F) phosphorylation at the tumor-ablation margin (denoted by white arrowheads) in the laser-ablated tumor but minimal AKT (C) and ERK (E) phosphorylation in the tumor, background liver or at the tumor-liver margin (denoted by white arrowheads) in the sham-ablated tumor. (*) denotes background liver.
(TIF)

S9 Fig Effect of PI3K and PI3K/mTOR inhibition on heat stress induced AKT signaling and cytotoxicity in HCC cells. **(A)** N1S1 and AS30D cells were pre-treated for one hour with LY294002 (50μM), NVP-BEZ235 (0.5μM) or vehicle control (0.1% DMSO) followed by heat stress (45°C) or control (37°C) for 10 minutes. Immediately post-heat stress whole-cell lysates were subjected to western immunoblotting for phospho- and total AKT. β-actin was used as a loading control. Representative images from 2 independent experiments (**B, C)** N1S1 and AS30D cells pre-treated for one hour with LY294002 (10 μM), NVP-BEZ235 (0.1 μM) or vehicle control (0.1% DMSO) followed by heat stress (45°C) or control (37°C) for 10 minutes were assessed with WST-1 viability assay at 72 hours post-heat stress. Data were normalized to 37°C vehicle control and presented as mean±SD (N = 4 independent cultures) (One-way ANOVA followed by post-hoc pairwise comparison using an unpaired t-test).
(TIF)

S10 Fig Effect of PI3K/mTORC1/2, AKT and mTORC1 inhibition on clonogenic survival to sublethal heat stress in Hep3B HCC cells. Human Hep3B HCC cells pre-treated with a dose-titration of the (A) the dual PI3K/mTORC1/2 inhibitor NVP-BEZ235, (B) the AKT inhibitor MK-2206 (C,) the mTORC1 inhibitor Rapamycin or vehicle control (0.1% DMSO) followed by heat stress (45°C) or control (37°C) for 10 minutes were plated in soft-agar and colonies of 50 or more cells were counted 14 days after plating using a light microscope. Percent colony formation per cell number plated was calculated and the data normalized to the non-heat stressed, 37°C control to calculate percent colony formation relative control to 37°C control. Data are presented as mean±SEM of 3 independent experiments (One-way ANOVA followed by post-hoc pairwise comparison using an unpaired t-test). ns P > 0.05; * P ≤ 0.05; ** P ≤ 0.01; *** P ≤ 0.001; **** P ≤ 0.0001
(TIFF)

S11 Fig Isoform-specific expression of AKT in rat and human HCC cells. Whole cell lysates were prepared from N1S1 and AS30D rat HCC cells and Hep3B and HuH7 human HCC cells and were subjected to western immunoblotting for AKT1, AKT2, AKT3 and total AKT. β-actin was used as a loading control.
(TIF)

S1 Table Doubling time and metabolic activity of Clone9 rat hepatocyte and N1S1 and AS30D rat HCC cell lines *in vitro*. (DOCX)

S2 Table Top Biological Functions: HCC v. Hepatocyte (Ingenuity Pathway Analysis). (DOCX)

S3 Table Top Canonical Pathways: HCC v. Hepatocyte (Ingenuity Pathway Analysis). (DOCX)

S4 Table Top Transcription Factors: HCC v. Hepatocyte (Ingenuity Pathway Analysis). (DOCX)

S5 Table Top Up- and Down-Regulated Molecules: HCC v. Hepatocyte (Ingenuity Pathway Analysis). (DOCX)

S6 Table *In vitro* and *in vivo* characteristics of Clone 9 hepatocyte and N1S1 and AS30D HCC model system. (DOC)

S1 File Whole transcriptome gene expression data for the N1S1 HCC versus Clone 9 hepatocyte cell lines. (XLSX)

S2 File Whole transcriptome gene expression data for the AS30D HCC versus Clone 9 hepatocyte cell lines. (XLSX)

S3 File Supplemental Methods. (DOCX)
