# Supplementary figures and images for: Heat Stress-Induced PI3K/mTORC2-Dependent AKT Signaling Is a Central Mediator of Hepatocellular Carcinoma Survival to Thermal Ablation Induced Heat Stress
Source: PLoS One. 2016 Sep 9;11(9):e0162634. doi: 10.1371/journal.pone.0162634 (PMC5017586; doi:10.1371/journal.pone.0162634)

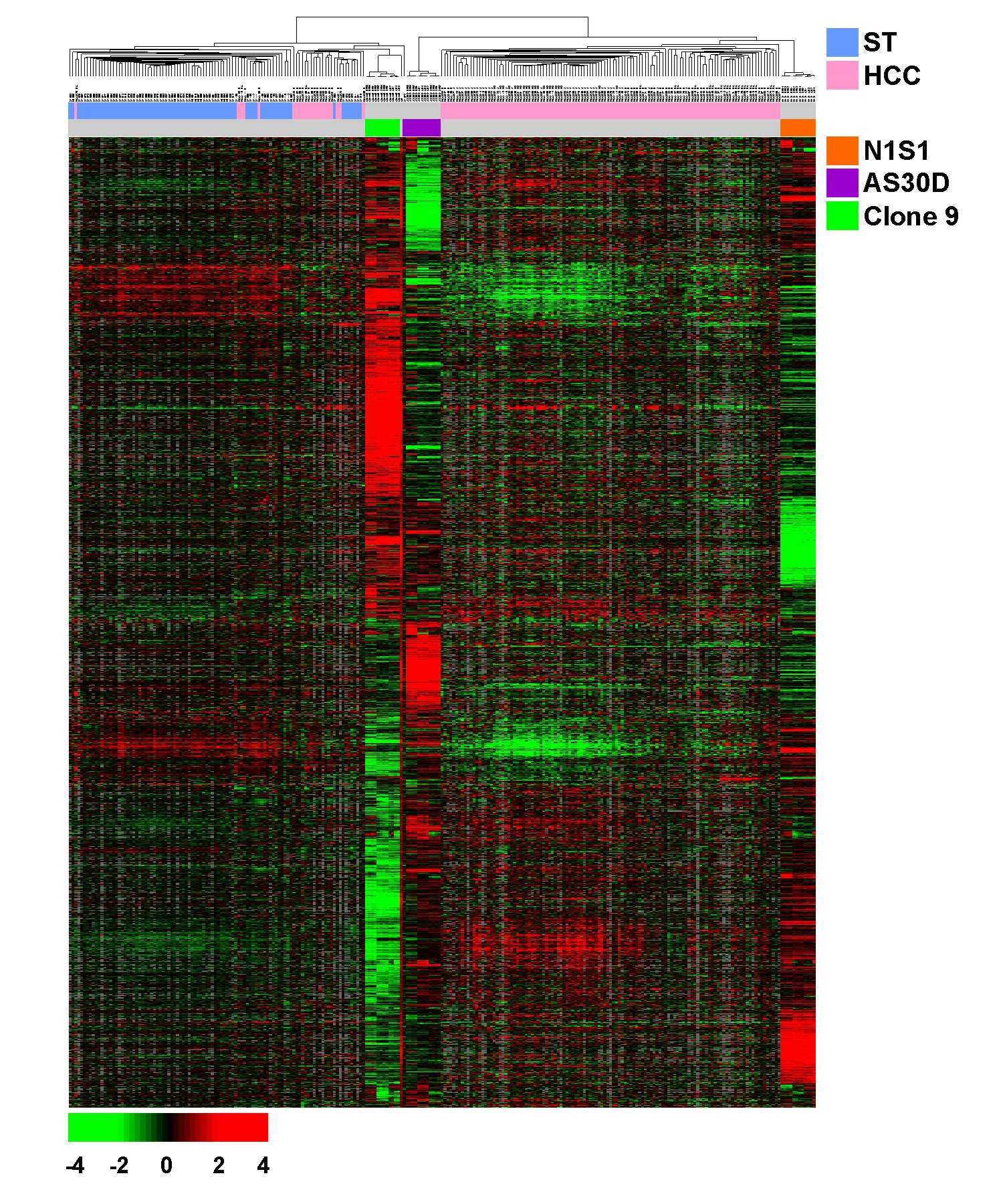

Supplement: S1 Fig — Hierarchical clustering analysis of integrated gene expression data from rat cell lines and human HCC tissues. Gene expression data from rat cell lines and human HCC tissues were independently centralized across samples to remove baseline differences between the two data sets. Genes with an expression level at least 2-fold different relative to the median value across tissues in at least 3 tissues in the rat data set were selected for hierarchical clustering analysis (2,759 unique gene). The data are presented in matrix format in which rows represent individual gene and columns represent each tissue. Each cell in the matrix represents the expression level of a gene feature in an individual tissue. The red and green color in cells reflect relative high and low expression levels respectively as indicated in the scale bar (log2 transformed scale). ST = BL = Benign Liver. HCC = Hepatocellular Carcinoma. (TIFF) [file pone.0162634.s001.tiff]

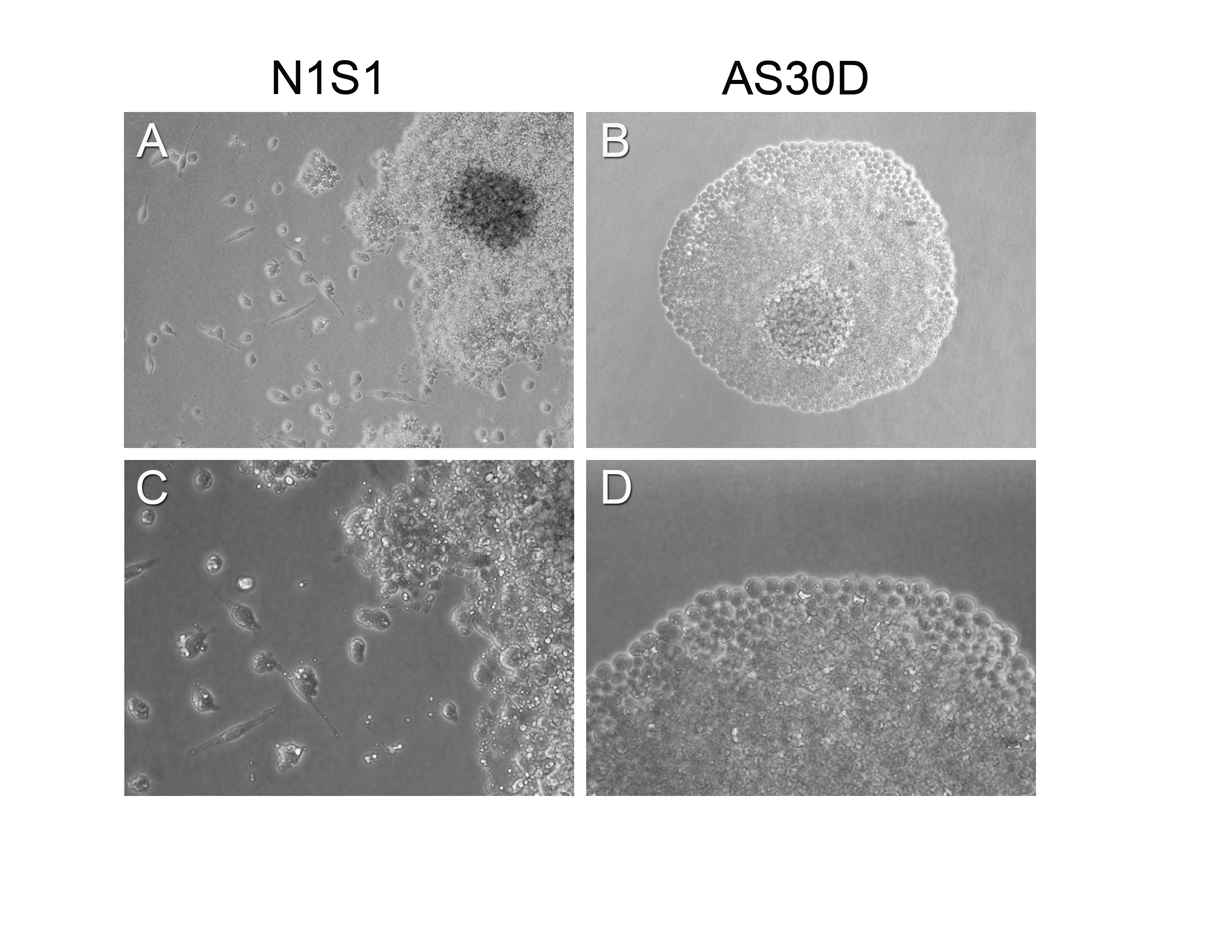

Supplement: S2 Fig — Representative photomicrographs of N1S1 (A,C) and AS30D (B,D) colonies after 10 days at 100x (A,B) and 200x (C,D) magnification. (TIF) [file pone.0162634.s002.tif]

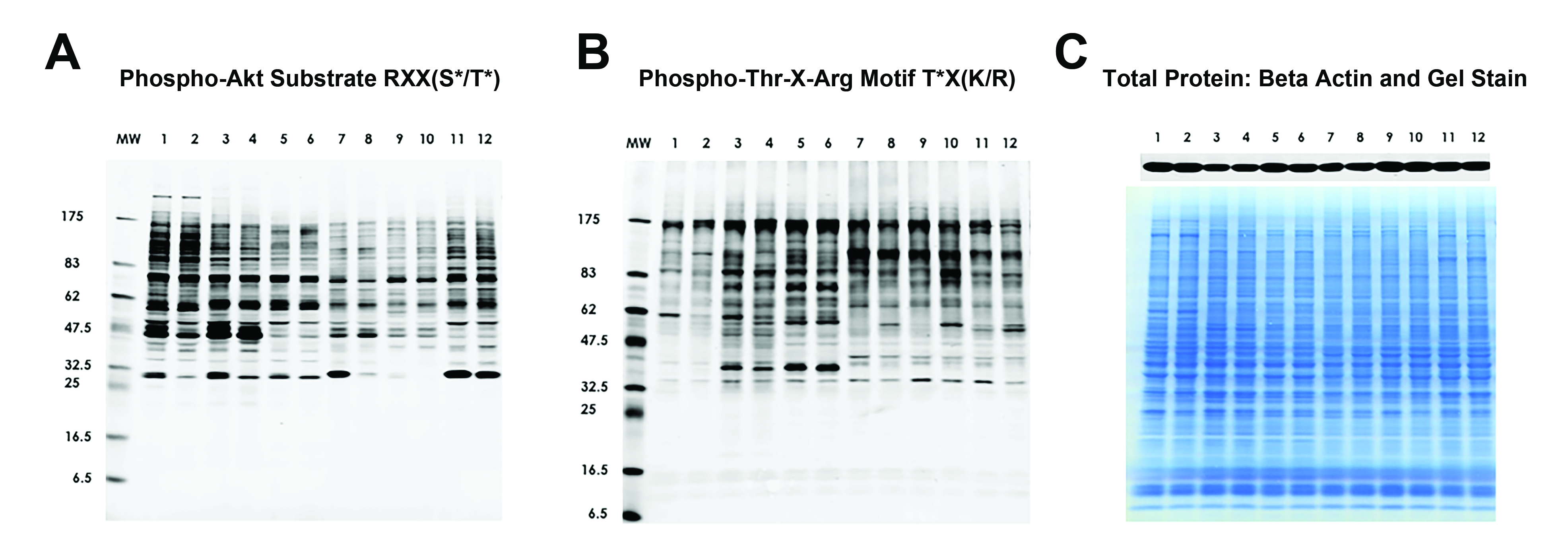

Supplement: S3 Fig — (A) Phospho-AKT Substrate RXX(S*T*) immunoblot; (B) Phospho-Thr-X-Arg Motif T*X(K/R). (C) β-Actin (top) and Gel Stain (bottom) control immunoblots. Odd numbered lanes are without heat stress and even numbered lanes are with heat stress. Lanes 1,2 = Clone rat hepatocyte; Lanes 3,4 = N1S1 rat HCC; Lanes 5,6 = AS30D rat HCC; Lane 7,8 = HuH7 human HCC; Lanes 9,10 = Hep3B human HCC; and Lanes 11,12 = PLC/PRF/5 human HCC. (TIF) [file pone.0162634.s003.tif]

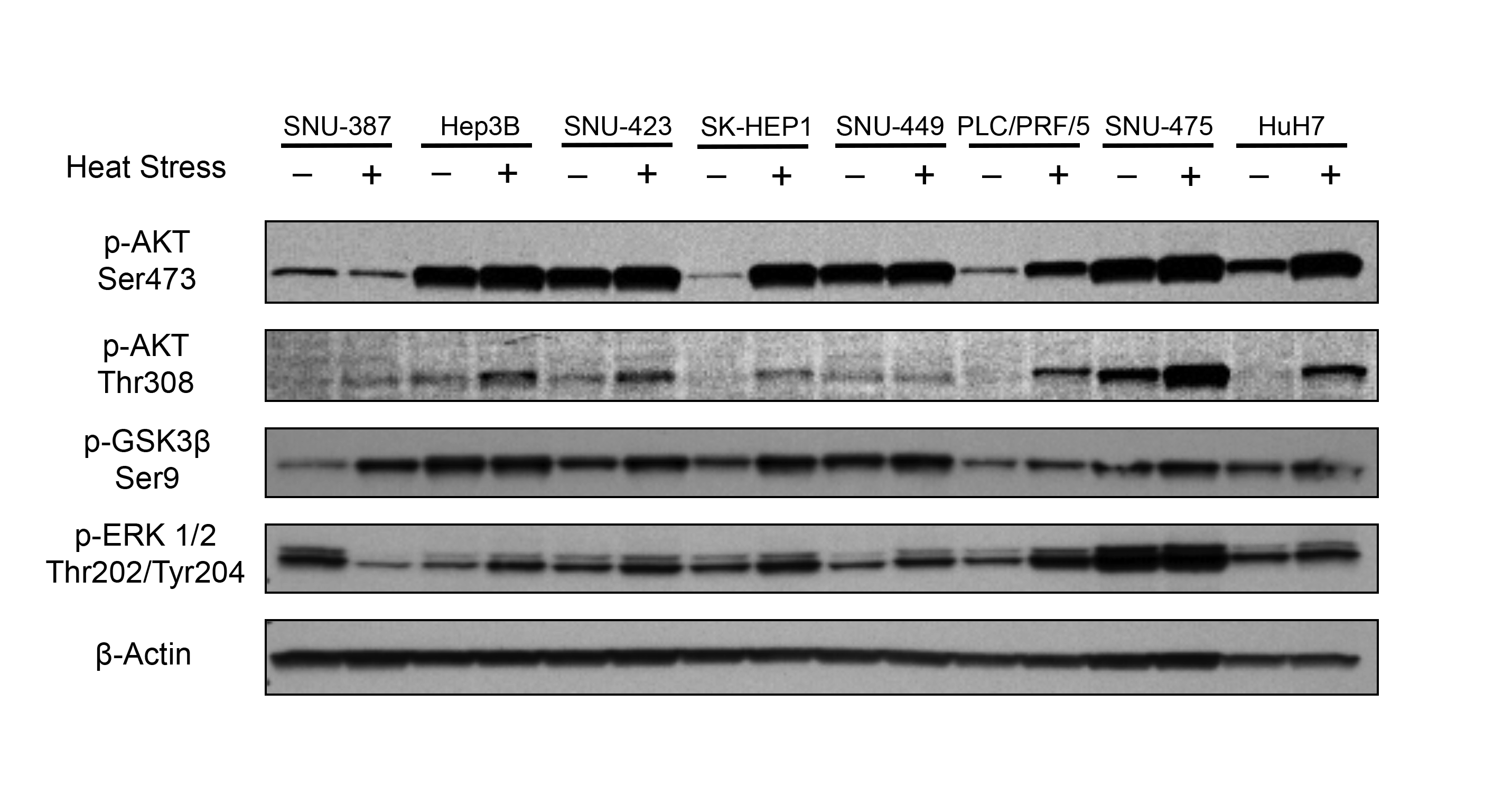

Supplement: S4 Fig — The indicated cell lines were heat stressed (45°C) or control (37°C) for 10 minutes, harvested immediately post-heat stress and whole-cell lysates were subjected to western immunoblotting using phospho-specific antibodies against AKT, GSK3β and ERK. β-actin was used as a loading control. (TIF) [file pone.0162634.s004.tif]

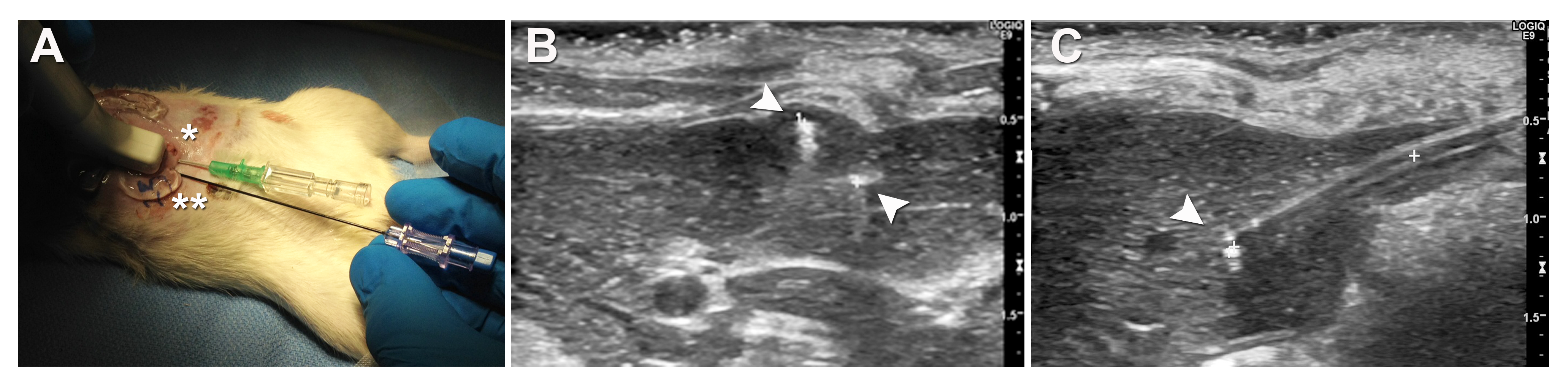

Supplement: S5 Fig — A) Percutaneous insertion of laser fiber (*) and thermocouple (**) into N1S1 tumor under US guidance. B) Short axis and C) long axis US images demonstrate a hypoechoic tumor (white arrowheads) with the hyperechoic-appearing laser fiber and thermocouple located in the inferior-lateral and superior-medial aspects of the tumor, respectively. (TIF) [file pone.0162634.s005.tif]

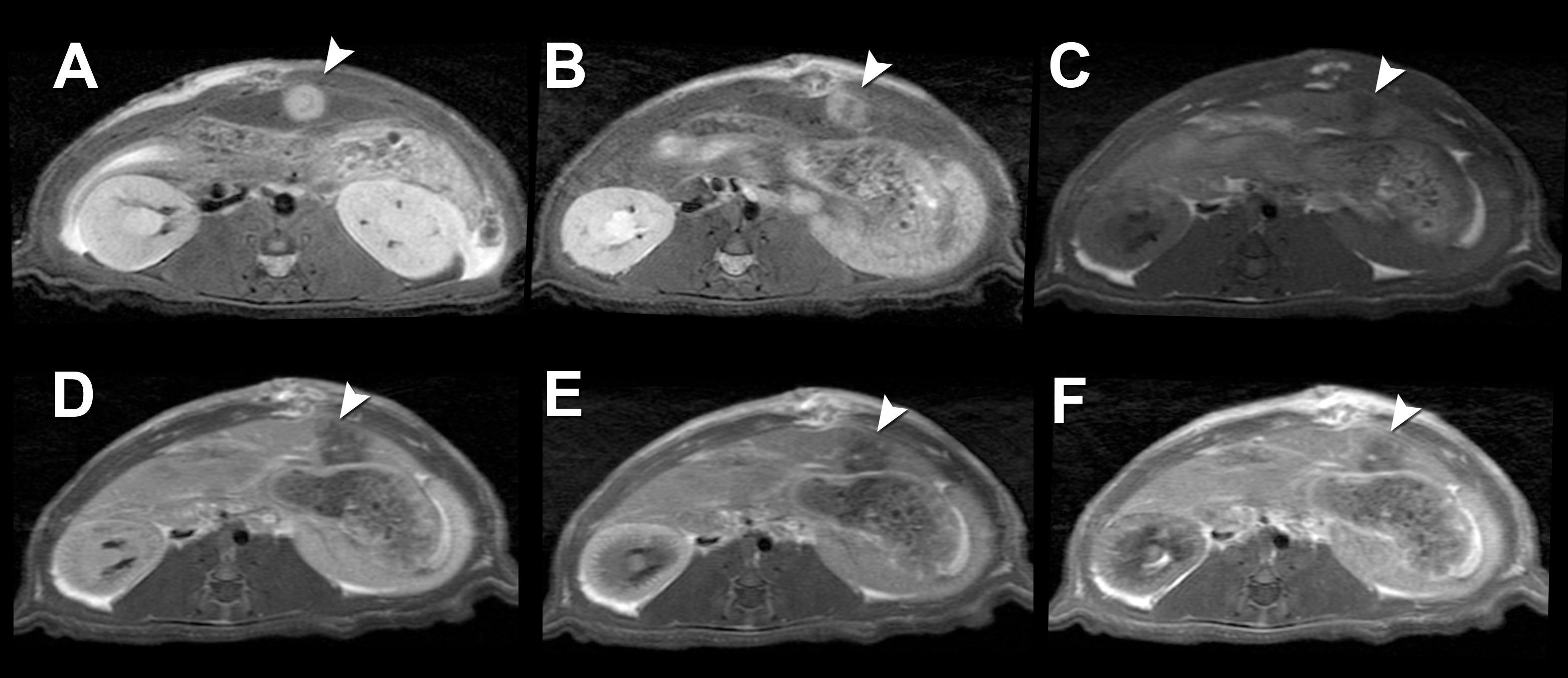

Supplement: S6 Fig — A) Pre-ablation and B-F) post-ablation 3T MR images demonstrate A) a hyperintense T2-weighted N1S1 tumor pre-ablation (denoted by white arrowhead), B) decreased T2-signal and C) decreased T1-signal relative to liver on immediate post-ablation non-contrast enhanced MRI. Gadolinium-enhanced T1-weighted MR images at D) 3-minutes E) 6-minutes and F) 10 minutes post-injection demonstrate time-dependent enhancement of the background liver and a hypoenhancing zone in the region of the tumor ablation. (TIF) [file pone.0162634.s006.tif]

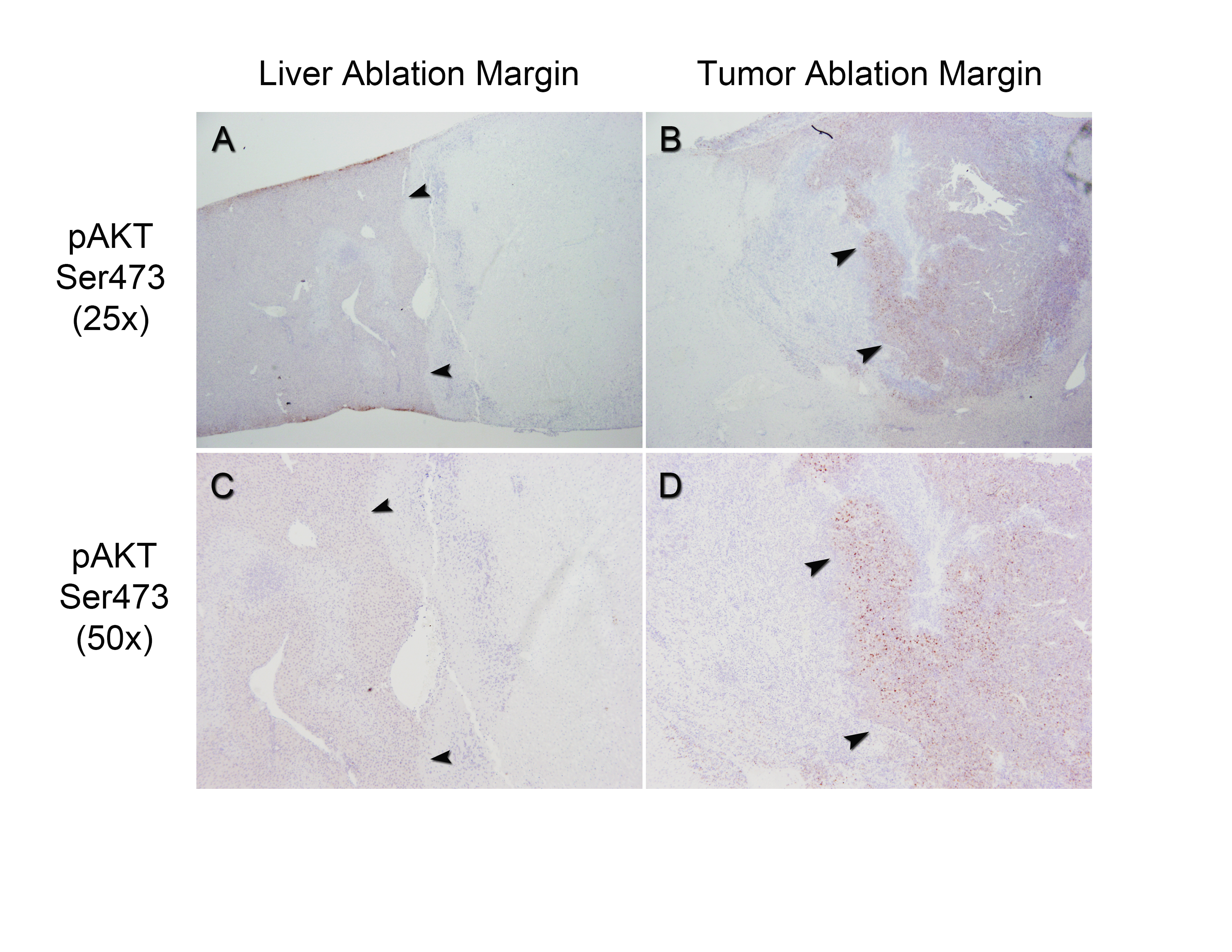

Supplement: S7 Fig — A) low power (25x) and C) higher power (50x) photomicrographs demonstrate very few cells staining positive (brown) for phospho-AKT in the background liver or at the liver-ablation margin (denoted by black arrowheads). B) low power (25x) and D) higher power (50x) photomicrographs demonstrate focal areas of markedly increased phospho-AKT immunostaining at the tumor ablation margin (denoted by black arrowheads) with decreased immunostaining further from the ablation margin toward the non-ablated tumor. (TIF) [file pone.0162634.s007.tif]

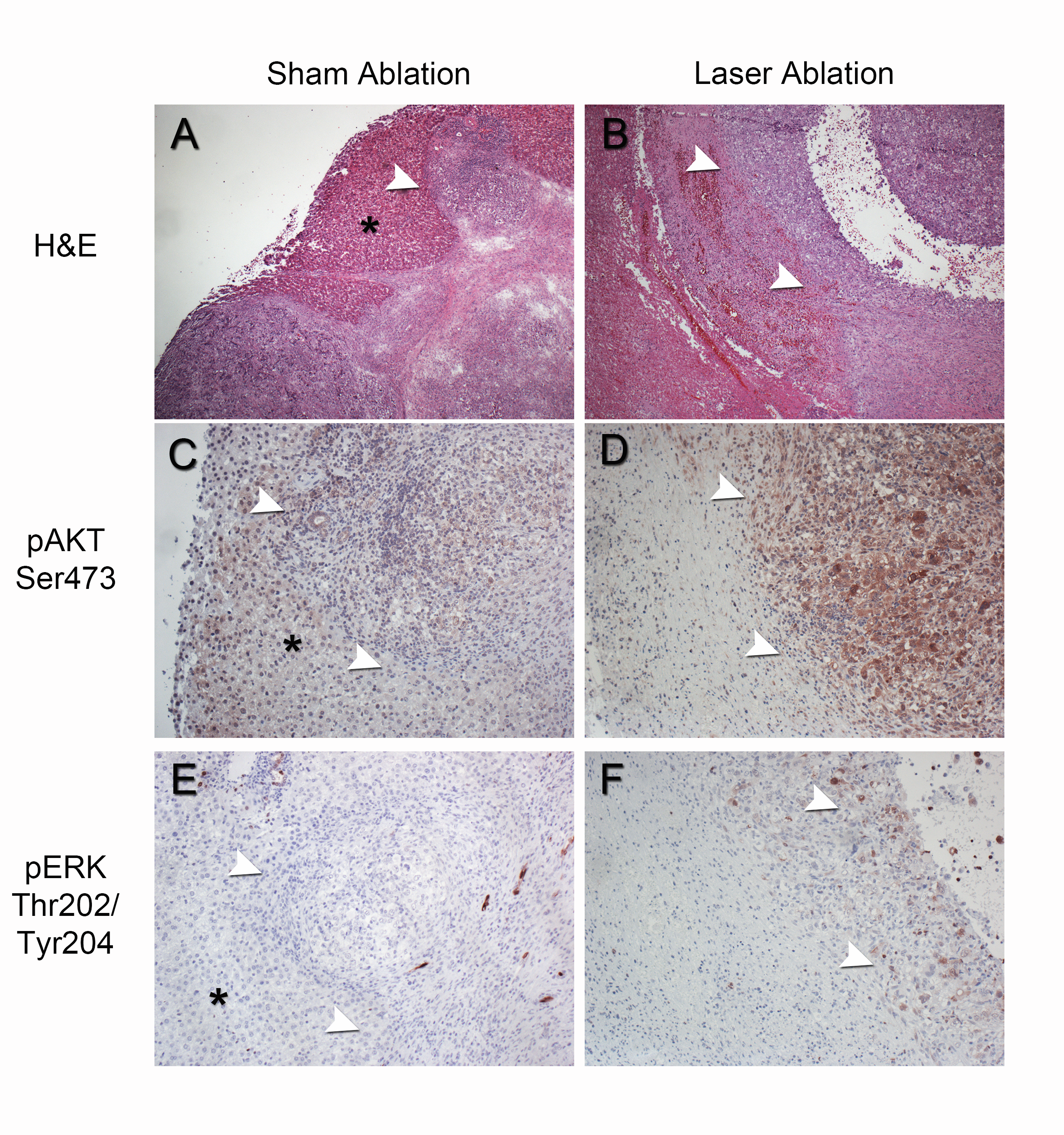

Supplement: S8 Fig — Photomicrographs (100x) of H&E stained sections (A,B) demonstrate A) the liver-tumor margin of a sham-ablated tumor (denoted by white arrowhead) and the B) tumor-ablation margin of a laser ablated tumor (denoted by white arrowheads). Corresponding photomicrographs (100x) of p-AKT (C,D) and p-ERK (E,F) immunostained sections demonstrate markedly increased AKT (D) and minimally increased ERK (F) phosphorylation at the tumor-ablation margin (denoted by white arrowheads) in the laser-ablated tumor but minimal AKT (C) and ERK (E) phosphorylation in the tumor, background liver or at the tumor-liver margin (denoted by white arrowheads) in the sham-ablated tumor. (*) denotes background liver. (TIF) [file pone.0162634.s008.tif]

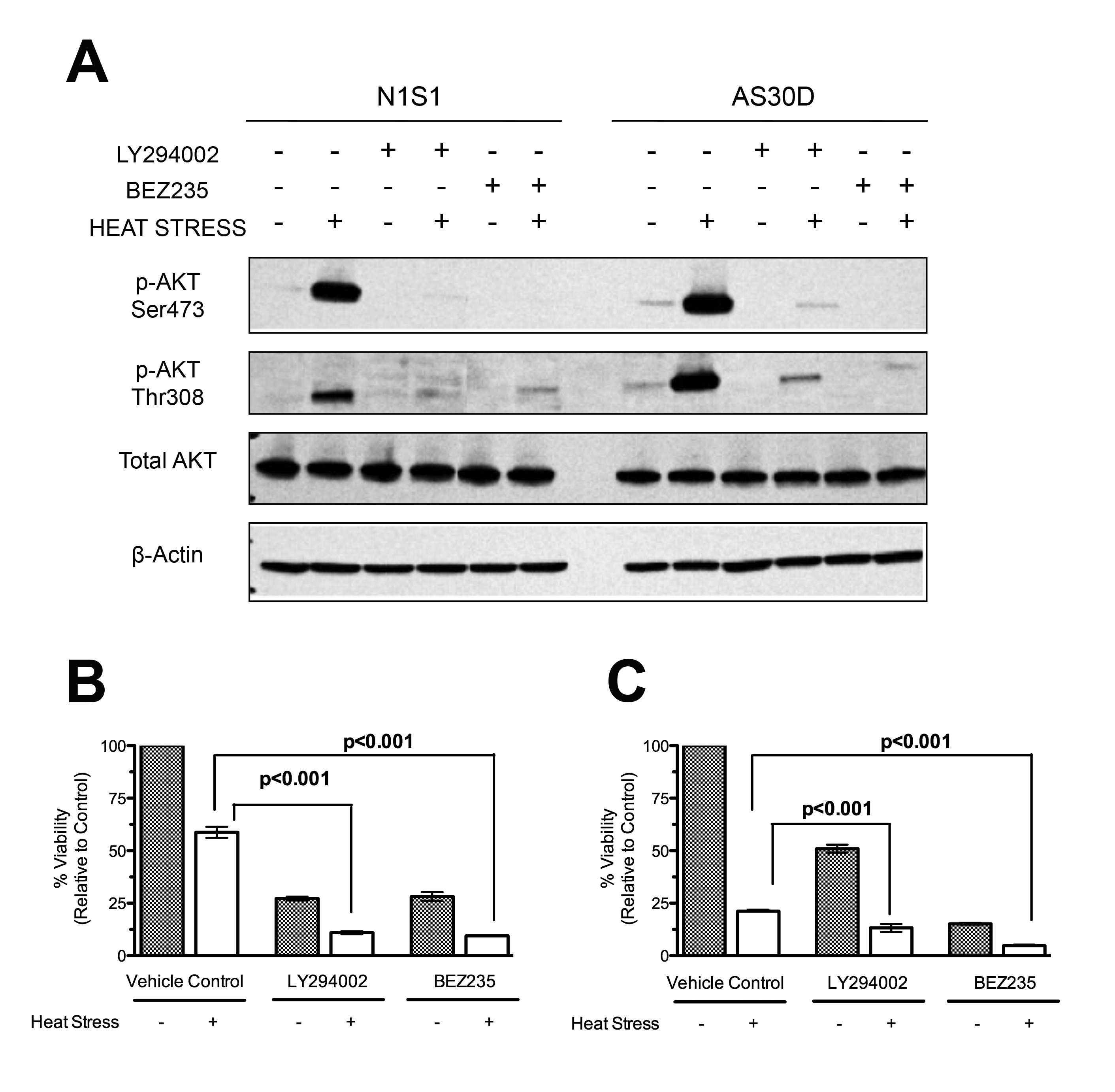

Supplement: S9 Fig — (A) N1S1 and AS30D cells were pre-treated for one hour with LY294002 (50μM), NVP-BEZ235 (0.5μM) or vehicle control (0.1% DMSO) followed by heat stress (45°C) or control (37°C) for 10 minutes. Immediately post-heat stress whole-cell lysates were subjected to western immunoblotting for phospho- and total AKT. β-actin was used as a loading control. Representative images from 2 independent experiments (B, C) N1S1 and AS30D cells pre-treated for one hour with LY294002 (10 μM), NVP-BEZ235 (0.1 μM) or vehicle control (0.1% DMSO) followed by heat stress (45°C) or control (37°C) for 10 minutes were assessed with WST-1 viability assay at 72 hours post-heat stress. Data were normalized to 37°C vehicle control and presented as mean±SD (N = 4 independent cultures) (One-way ANOVA followed by post-hoc pairwise comparison using an unpaired t-test). (TIF) [file pone.0162634.s009.tif]

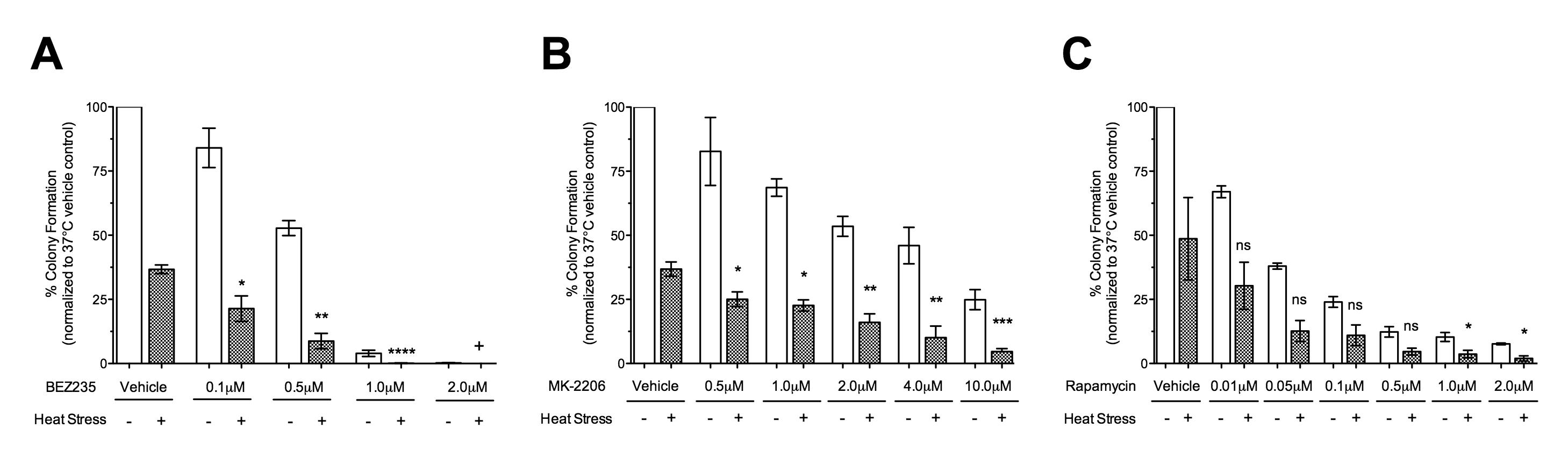

Supplement: S10 Fig — Human Hep3B HCC cells pre-treated with a dose-titration of the (A) the dual PI3K/mTORC1/2 inhibitor NVP-BEZ235, (B) the AKT inhibitor MK-2206 (C,) the mTORC1 inhibitor Rapamycin or vehicle control (0.1% DMSO) followed by heat stress (45°C) or control (37°C) for 10 minutes were plated in soft-agar and colonies of 50 or more cells were counted 14 days after plating using a light microscope. Percent colony formation per cell number plated was calculated and the data normalized to the non-heat stressed, 37°C control to calculate percent colony formation relative control to 37°C control. Data are presented as mean±SEM of 3 independent experiments (One-way ANOVA followed by post-hoc pairwise comparison using an unpaired t-test). ns P > 0.05; * P ≤ 0.05; ** P ≤ 0.01; *** P ≤ 0.001; **** P ≤ 0.0001 (TIFF) [file pone.0162634.s010.tiff]

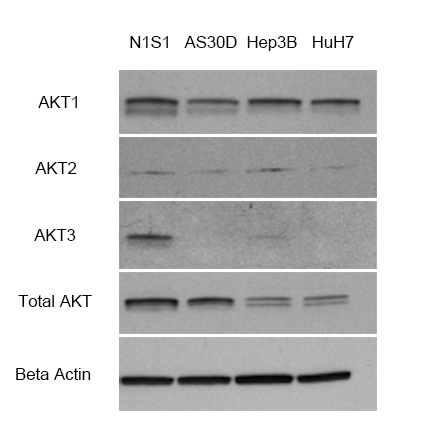

Supplement: S11 Fig — Whole cell lysates were prepared from N1S1 and AS30D rat HCC cells and Hep3B and HuH7 human HCC cells and were subjected to western immunoblotting for AKT1, AKT2, AKT3 and total AKT. β-actin was used as a loading control. (TIF) [file pone.0162634.s011.tif]
