# Supplementary material for: Heat Stress-Induced PI3K/mTORC2-Dependent AKT Signaling Is a Central Mediator of Hepatocellular Carcinoma Survival to Thermal Ablation Induced Heat Stress
Source: PLoS One. 2016 Sep 9;11(9):e0162634. doi: 10.1371/journal.pone.0162634 (PMC5017586; doi:10.1371/journal.pone.0162634)
Supplement: S1 Table — (DOCX) [file pone.0162634.s012.docx]

S1 Table. Doubling time and metabolic activity of Clone9 rat hepatocyte and N1S1 and AS30D rat HCC cell lines *in vitro*.

| **Cell Line** | **Doubling Time (hours)** | **Metabolic Activity**  **(uU/hour/cell)** |
| --- | --- | --- |
| **Clone9** | 24.7±2.1 | 0.55±0.05 |
| **N1S1** | 18.9±1.4 | 1.31±0.10 |
| **AS30D** | 33.2±5.3 | 0.97±0.16 |

Data are presented as mean±SD (N=6 independent cultures).
