# Supplementary material for: Heat Stress-Induced PI3K/mTORC2-Dependent AKT Signaling Is a Central Mediator of Hepatocellular Carcinoma Survival to Thermal Ablation Induced Heat Stress
Source: PLoS One. 2016 Sep 9;11(9):e0162634. doi: 10.1371/journal.pone.0162634 (PMC5017586; doi:10.1371/journal.pone.0162634)
Supplement: S2 Table — (DOCX) [file pone.0162634.s013.docx]

S2 Table: Top Biological Functions: HCC v. Hepatocyte (Ingenuity Pathway Analysis)

| **N1S1 HCC v. Clone9 Hepatocyte** | | | | **AS30D HCC v. Clone9 Hepatocyte** | | |
| --- | --- | --- | --- | --- | --- | --- |
|  |  | **p-value** | **# Molecules** |  | **p-value** | **# Molecules** |
| Diseases and Disorders | Cancer | 3.99E-28 - 3.11E-04 | 1326 | Cancer | 3.06E-33 - 7.70E-05 | 1262 |
|  | Gastrointestinal Disease | 1.00E-16 - 2.99E-04 | 1071 | Reproductive System Disease | 5.87E-16 - 5.08E-06 | 662 |
|  | Reproductive System Disease | 1.89E-10 - 3.11E-04 | 618 | Gastrointestinal Disease | 1.13E-14 - 7.31E-06 | 1051 |
|  | Infectious Disease | 3.01E-10 - 2.00E-04 | 490 | Endocrine System Disorders | 5.79E-12 - 7.49E-05 | 78 |
|  | Genetic Disorder | 4.93E-09 - 3.11E-04 | 782 | Metabolic Disease | 5.79E-12 - 4.08E-05 | 768 |
|  |  | **p-value** | **# Molecules** |  | **p-value** | **# Molecules** |
| Molecular and Cellular Functions | Cell Death | 3.01E-31 - 3.38E-04 | 1013 | Cell Death | 1.45E-36 - 8.81E-05 | 1040 |
|  | Cellular Growth and Proliferation | 3.39E-29 - 2.79E-04 | 1060 | Cellular Growth and Proliferation | 7.19E-33 - 8.46E-05 | 1094 |
|  | Gene Expression | 3.44E-21 - 7.20E-05 | 771 | Gene Expression | 4.70E-30 - 4.61E-05 | 811 |
|  | Cell Cycle | 1.27E-19 - 2.93 E-04 | 483 | Cellular Development | 1.75E-23 - 8.46E-05 | 833 |
|  | Cellular Development | 2.73E-19 - 2.76E-04 | 772 | Cell Cycle | 6.00E-20 - 8.61E-05 | 485 |
