# Supplementary material for: Heat Stress-Induced PI3K/mTORC2-Dependent AKT Signaling Is a Central Mediator of Hepatocellular Carcinoma Survival to Thermal Ablation Induced Heat Stress
Source: PLoS One. 2016 Sep 9;11(9):e0162634. doi: 10.1371/journal.pone.0162634 (PMC5017586; doi:10.1371/journal.pone.0162634)
Supplement: S3 Table — (DOCX) [file pone.0162634.s014.docx]

S3 Table: Top Canonical Pathways: HCC v. Hepatocyte (Ingenuity Pathway Analysis)

| **N1S1 HCC v. Clone9 Hepatocyte** | | | | **AS30D HCC v. Clone9 Hepatocyte** | | |
| --- | --- | --- | --- | --- | --- | --- |
|  |  | **p-value** | **Ratio** |  | **p-value** | **Ratio** |
| Canonical Pathways | Molecular Mechanisms of Cancer | 6.95E-11 | 120/379 (0.317) | Molecular Mechanisms of Cancer | 5.02E-11 | 121/379 (0.319) |
|  | Cell Cycle: G1/S Checkpoint Regulation | 6.99E-07 | 28/61 (0.459) | Role of Tissue Factor in Cancer | 5.02E-11 | 49/114 (0.43) |
|  | Pyrimidine Metabolism | 1.6E-06 | 53/217 (0.244 | PTEN Signaling | 1.44E-07 | 46/124 (0.371) |
|  | p53 Signaling | 1.67E-06 | 39/96 (0.406) | Glucocorticoid Receptor Signaling | 2.02E-07 | 89/295 (0.302) |
|  | Role of BRCA1 in DNA Damage Response | 2.72E-06 | 27/61 (0.443) | Small Cell Lung Cancer Signaling | 2.11E-07 | 34/89 (0.382) |
