# Supplementary material for: Heat Stress-Induced PI3K/mTORC2-Dependent AKT Signaling Is a Central Mediator of Hepatocellular Carcinoma Survival to Thermal Ablation Induced Heat Stress
Source: PLoS One. 2016 Sep 9;11(9):e0162634. doi: 10.1371/journal.pone.0162634 (PMC5017586; doi:10.1371/journal.pone.0162634)
Supplement: S4 Table — (DOCX) [file pone.0162634.s015.docx]

S4 Table: Top Transcription Factors: HCC v. Hepatocyte (Ingenuity Pathway Analysis)

| **N1S1 HCC v. Clone9 Hepatocyte** | | | | **AS30D HCC v. Clone9 Hepatocyte** | | |
| --- | --- | --- | --- | --- | --- | --- |
|  |  | **p-value** | **Predicted Activation Sate** |  | **p-value** | **Predicted Activation Sate** |
| Transcription Factors | TP53 | 1.38E-40 | Inhibited | TP53 | 6.09E-34 | Inhibited |
|  | MYC | 2.36E-26 | Activated | MYC | 3.98E-29 | Activated |
|  | HNF4A | 1.84E-25 | - | HNF4A | 1.09E-23 | - |
|  | AHR | 1.59E-12 | - | NR3C1 | 5.93E-18 | - |
|  | FOS | 1.24E-11 | Inhibited | CTNNB1 | 1.20E-13 | Inhibited |
