# Supplementary material for: Heat Stress-Induced PI3K/mTORC2-Dependent AKT Signaling Is a Central Mediator of Hepatocellular Carcinoma Survival to Thermal Ablation Induced Heat Stress
Source: PLoS One. 2016 Sep 9;11(9):e0162634. doi: 10.1371/journal.pone.0162634 (PMC5017586; doi:10.1371/journal.pone.0162634)
Supplement: S5 Table — (DOCX) [file pone.0162634.s016.docx]

S5 Table: Top Up- and Down-Regulated Molecules: HCC v. Hepatocyte (Ingenuity Pathway Analysis)

|  | **N1S1 HCC v. Clone9 Hepatocyte** | | **AS30D HCC v. Clone9 Hepatocyte** | |
| --- | --- | --- | --- | --- |
|  | **Molecule** | **Fold-Change** | **Molecule** | **Fold-Change** |
| Fold Change up-regulated | PTGR1 | 2865.6 | AGR2 | 2760.7 |
|  | KCNAB1 | 1608.2 | 1600029D21Rik | 2667.4 |
|  | RPS4Y1 | 1441.3 | LGALS4 | 2083.8 |
|  | CLCA4 | 952.5 | Cd24a | 1781.5 |
|  | PCSK9 | 842.4 | CDH17 | 1560.8 |
|  | ELAVL2 | 811.6 | PCSK9 | 1466.8 |
|  | SLC14A1 | 803.0 | Krt19 | 1312.6 |
|  | NXPH1 | 751.9 | PCBD1 | 842.1 |
|  | AQP8 | 728.6 | FABP4 | 707.3 |
|  | PEM | 655.0 | PEM | 607.8 |
| Fold Change down-regulated | CTGF | -4071.3 | COL1A2 | -4133.3 |
|  | COL1A2 | -3705.1 | CD81 | -3954.8 |
|  | PRSS23 | -3554.3 | COL3A1 | -3421.8 |
|  | LOX | -2377.1 | TM4SF1 | -3196.0 |
|  | COL3A1 | -2367.6 | LOX | -2362.3 |
|  | COL5A2 | -2061.7 | CCDC80 | -2206.7 |
|  | CCDC80 | -1906.5 | COL5A2 | -2151.7 |
|  | MGP | -1795.4 | CAV1 | -2007.3 |
|  | COL1A1 | -1698.2 | COL1A1 | -1984.9 |
|  | GPC3 | -1649.6 | F3 | -1979.9 |
