# Supplementary material for: Heat Stress-Induced PI3K/mTORC2-Dependent AKT Signaling Is a Central Mediator of Hepatocellular Carcinoma Survival to Thermal Ablation Induced Heat Stress
Source: PLoS One. 2016 Sep 9;11(9):e0162634. doi: 10.1371/journal.pone.0162634 (PMC5017586; doi:10.1371/journal.pone.0162634)
Supplement: S6 Table — (DOC) [file pone.0162634.s017.doc]

S6 Table. *In vitro* and *in vivo* characteristics of Clone 9 hepatocyte and N1S1 and AS30D HCC model system

| **Cell Line** | **Clone9 Hepatocyte** | **N1S1**  **HCC** | **AS30D**  **HCC** |
| --- | --- | --- | --- |
| *In vitro* |  |  |  |
| **Proliferation Doubling Time** | Intermediate  25hr | Fast  19hr | Slow  32hr |
| **Growth** | Monolayer | Spheroid Suspension | Single-cell  Suspension |
| **Colony Formation**  **(soft agar)** | No | Yes | Yes |
| **Migration**  **(soft agar)** | No | Yes | No |
| **Molecular Subtype** | Normal Liver | Hepatic Stem Cell HCC | Well Differentiated HCC |
| **Prognostic Subtype** | - | Poor | Better |
| *In vivo* |  |  |  |
| **Tumorigenic** | No | Yes | Yes |
| **Growth** | - | Rapid | Slower |
