# Supplementary material for: Heat Stress-Induced PI3K/mTORC2-Dependent AKT Signaling Is a Central Mediator of Hepatocellular Carcinoma Survival to Thermal Ablation Induced Heat Stress
Source: PLoS One. 2016 Sep 9;11(9):e0162634. doi: 10.1371/journal.pone.0162634 (PMC5017586; doi:10.1371/journal.pone.0162634)
Supplement: S3 File — (DOCX) [file pone.0162634.s020.docx]

**SUPPLEMENTAL METHODS**

**Whole Transcriptome Gene Expression Analysis**

Data pre-processing and normalization: The individual mRNA expression profile was calculated for each probe using MAPP. MAPP normalizes the data and eliminates background noise present in the raw data as well. After running the MAPP software, the samples were separated based on the groups used for comparison. Quality control analysis: Principle Component Analysis (PCA) plot and hierarchical clustering from Partek are plotted using the log2 transformed data, obtained from MAPP, to test the quality of the dataset. Low / unexpressed probe filtering: After normalization and pre-processing by MAPP, probes that were not present in any of the samples were disregarded. Differentially expressed genes: p-values for each probe were calculated using t-test and probes with p-value > 0.05 were filtered out. Additional filtering of Fold-Change |1.5| and Fold-Change |2| were also calculated. Pathway analysis: Ingenuity was used to perform pathway analysis and identify significant pathways affected by the differentially expressed genes. The probes with p<0.05 and |FC|>=2 were used as input for the analysis.

**Continuous equivalent minutes at 43°C (CEM43)**

Because thermal dose is a function of both temperature and exposure time, a normalized thermal dose known as cumulative equivalent minutes at 43°C (CEM43) can be calculated based on temperature and exposure time data according to the method by Saparento et al [1].

(1) CEM43 = t x R^(43-T)^

t = exposure time (min)

T = temperature (°C)

R = 0.5 (T>43°C); 0.25 (T<43°C)

Heat stress exposure time (min) and temperature (°C) data were converted to CEM43, log_10_ transformed and plotted versus viability. An IT_50_ for CEM43, heating time in minutes at 43°C to achieve a 50% kill, was calculated using non-linear regression curve fitting as described above.

1. Sapareto SA, Dewey WC. Thermal dose determination in cancer therapy. Int J Radiat Oncol Biol Phys. 1984;10(6):787-800.

Description of Kinomeview® antibodies and target consensus site

| **Description** | **Antibody** | **Consensus Site** |
| --- | --- | --- |
| Phospho-Akt Substrate | 9614 | RXX(S*/T*) |
| Phospho-Akt Substrate | 10001 | RXRXX(S*/T*) |
| Phospho-(Ser/Thr) AMPK Substrate | 5759 | (L/M)XRXX(S*/T*), RXX(S*/T*) |
| Phospho-(Ser) ATM/ATR Substrate | 9607 | S*Q |
| Phospho-(Ser/Thr) ATM/ATR Substrate | 6966 | (S*/T*)QG, (S*/T*)Q |
| Phospho-(Ser) CDKs Substrate | 9477 | (K/H)S*P |
| Phospho-(Ser/Thr) CK II Substrate | 8738 | (S*/T*)DXE |
| Phospho-MAPK/CDK Substrates | 2325 | PXS*P, S*PX(K/R) |
| Phospho-(Ser/Thr) PDK1 Docking Motif | 9634 | (F/K)XX(F/Y)(S*/T*)(F/Y) |
| Phospho-PKA Substrate | 9624 | (K/R)(K/R)X(S*/T*) |
| Phospho-(Ser) PKC Substrate | 6967 | (K/R)XS*X(K/R) |
| Phospho-(Thr) PLK Binding Motif | 5243 | ST*P |
| Phospho-Thr-Pro Motif | 3003 | T*P, T*PP |
| Phospho-Thr-Pro-Glu Motif | 3004 | T*PE, T*P |
| Phospho-Thr-X-Arg Motif | 2351 | T*X(K/R) |
| Phospho-Tyrosine (P-Tyr-100) | 9411 | Y* |
| Ubiquitin Branch | 3925 | K-ε-GG |
| Acetylated-Lysine | 9814 | Ac-K |
| Mono-Methylated-Arginine | 8015 | Me-R |
| Mono-Methylated-Arginine | 8711 | Me-RGG |
